# Mathematical formulations and a Relax-and-Fix heuristic algorithm for capacitated reliable fixed-charge facility location problems

**DOI:** 10.1007/s10732-025-09577-y

**Published:** 2025-12-29

**Authors:** Abdolreza Roshani, Glenn Parry, Philip Walker-Davies

**Affiliations:** 1 Centre for Business Analytics in Practise, Surrey Business School, University of Surrey, Guildford, UK; 2 Centre of Digital Economy, Surrey Business School, University of Surrey, Guildford, UK; 3 University of Bristol Business School, University of Bristol, Bristol, UK

**Keywords:** Capacity, Reliable facility location, Supply Chain Disruption, Optimisation, Relax-and-Fix heuristic

## Abstract

The reliable fixed-charge facility location problem extends the fixed-cost facility location problem by incorporating facility unreliability. This paper addresses a novel capacitated version of the reliable fixed-charge facility location problem, where the failure probability of each facility is site-dependent, differing from existing literature models. Additionally, facilities are assumed to have limited capacity for demand allocation, with the option to increase capacity to a predetermined value in case of supply chain disruptions. To solve this problem, we develop a non-linear mixed-integer programming formulation and present its linear version. Given the NP-hard nature of the problem, we propose a novel Relax-and-Fix heuristic for its solution. We evaluate the efficiency of the proposed algorithm by solving a variety of experimental instances with different network sizes. Results demonstrate that the Relax-and-Fix heuristic improves upper bounds for problem instances and achieves this within a shorter computational time. Furthermore, sensitivity analysis is conducted on capacity and failure probabilities, and relevant results are presented.

## Introduction

Natural disasters such as the COVID-19 pandemic, floods, hurricanes, and man-made threats such as war and cyber-attacks have an unprecedented and for many unexpected impact on global supply chains (SC). Although the frequency of their occurrence may be low, such disasters may result in significant production disruption and delivery delays (Katsoras and Georgiadis 2022). For example, some Foxconn facilities in China were forced to close as a result of the Wuhan lockdown during the outbreak of COVID-19, causing Apple to postpone the market release of new products (Xu et al. 2020; Ye et al. 2022). The tsunami and earthquake that struck Japan in 2011 forced Toyota and General Motors to halt production for several days since their suppliers were unable to deliver parts and raw materials at the expected volume and time (Huffington 2015; Hosseini et al. 2019). To mitigate the negative impact of such disruptive events, designing resilient supply chains that can quickly adjust to disruptive changes that negatively affect performance (Wieland and Durach 2021) is thus a key goal for firms (Ivanov et al. 2017; Aldrighetti et al. 2021; Roshani et al. 2024). This is particularly important given the effects of climate change and cyber-attacks are expected to become increasingly frequent and more severe (Ghadge et al. 2020; Katsoras and Georgiadis 2022).

Supply Chain Network Design (SCND) is an operations research discipline that aims to identify the optimal configuration for facilities and the flow of goods within a supply chain. It encompasses a range of activities, including the strategic determination of facility locations and sizes, as well as the optimization of goods movement throughout the network (Farahani et al. 2014). The extensive literature on SCND offers various models that support decision-making processes. Among these decisions, the strategic placement of facilities at different tiers within the supply chain emerges as one of the most critical factors which significantly influences the overall performance and cost-effectiveness of the entire supply chain (Farahani et al. 2014; Eskandarpour et al. 2015).

The reliable p-median and fixed-charge facility location problems denoted as RPMP and RFLP respectively, are generalised versions of the classical p-median and fixed-charged facility location problems. The p-median problem (PMP) determines the optimal locations for a given number of facilities in a network to minimize total weighted distance/cost of serving all the demand nodes (Church and Wang 2020). The fixed-charge facility location problem (FLP) chooses facility locations and assigns customers to facilities to minimize the sum of fixed and transportation costs (Klose and Drexl 2005). The main difference between reliable and classical versions of PMP and FLP is that, in reliable versions, it is assumed that one or more facilities may become unavailable from time to time due to disruptive events (Snyder and Daskin 2005). If a facility fails, the demand of the customers assigned to it must be covered by other facilities (which are probably further away from the primary facility), which imposes a higher transportation cost on the system. Thus, RPMP and RFLP aim to increase the reliability and resiliency of a supply chain in case of disruptive events by suggesting an appropriate design for its network (Aydin and Murat 2013; Snyder et al. 2016). In this article, we focus on the RFLP.

Taking into account the capacity constraint for facilities, RFLP can be categorised into two groups: uncapacitated and capacitated problems. Reliable uncapacitated FLP (RUFLP) assumes that each facility has an unbounded capacity while in reliable capacitated FLP (RCFLP) the capacities are limited. RUFLP has been the focus of many researchers and has been well-studied in the literature (Snyder et al. 2016; Hosseini et al. 2019; Aldrighetti et al. 2021). Snyder and Daskin (2005) were among the first researchers to study this problem and they defined reliability in the context of facility location problems as the ability of a system to perform well even when parts of the system have failed. Two classes of facility location problems were studied, namely, p-median and FLP. By assuming that the failure probabilities of facilities were identical, Snyder and Daskin (2005) proposed mathematical formulations and Lagrangian relaxation (LR) algorithms to choose facility locations that are both inexpensive and reliable. Lim et al. (2010) addressed a class of RUFLP which incorporated two types of facilities, one that is unreliable and another that is reliable. They assumed that a reliable facility is more expensive but not subject to disruption. The problem was formulated as a mixed integer programming model and a LR-based solution algorithm was developed to solve the problem. Cui et al. (2010) proposed a mixed integer program (MIP) formulation, LR algorithm and a continuum approximation (CA) model for RUFLP considering unexpected failures with site-dependent probabilities and possible customer reassignment to a limited number of facilities. Alcaraz et al. (2012) addressed the Reliable p-Median Problem (RPMP) and presented a genetic algorithm and a scatter search approach to solve the problem. Aboolian et al. (2013) extended Cui et al. (2010) problem to a class of RUFLP in which the number of facilities assigned to a customer is not limited. By combining neighborhood search and cutting plane process, they proposed a novel approach, which outperformed Cui et al. (2010) in both execution time and solution quality, especially when a large number of facilities are allowed to be assigned to a customer. Li et al. (2013) proposed a mathematical programming model that determines the optimal network location design for an infrastructure system under correlated facility failure risks. Alcaraz et al. (2015) formulated the original mathematical programming model of the RUFLP proposed by Snyder and Daskin (2005) as a set packing problem and study some conditions for optimal solutions. Yu and Zhang (2018) consider risk-averse RUFLP and used conditional-value-at-risk to control the risks for each individual customer. A mixed-integer nonlinear programming formulation and a multi-dual decomposition algorithm based on the augmented LR and classic penalty function were proposed.

A few researchers also addressed RCFLP. Aydin and Murat (2013) proposed a scenario-based formulation for RCFLP as a two-stage stochastic program. They presented a novel hybrid method, swarm intelligence-based sample average approximation, for solving the problem. Albareda-Sambola et al. (2017) addressed a version of RCFLP in which capacity constraints on the facilities were stated as hard constraints for the scenario where no failures occurred, but relatively small violations were allowed if they did. They also assumed that facilities could fail with homogeneous probability, and these failures occurred independently. Mathematical models were defined and tested based on the definition of different bounds and capacity constraints. Azad and Hassini (2019) studied RCFLP with the aim of investigating different mitigation strategies, and partial failures. A mixed-integer linear programming model was proposed for the problem and an accelerated Banders’ decomposition method was used to solve it. Cheng et al. (2021) studied capacitated fixed-charge location problem (CFLP) in which customers are subject to demand uncertainty and facilities may experience disruption risks. The authors adopt a two-stage robust optimization (RO) scheme to model the problem and apply a column-and-constraint-generation (C&CG) algorithm based on a decomposition scheme.

By considering how to model the probability of facility failure, the mathematical formulations presented to solve both RUFLP and RCFLP can be categorised into five main categories (Lu et al. 2015; Snyder et al. 2016): nonlinear probability terms or implicit functions (IF) models, scenario-based (SB) models, reliable backups (RB) models, continuum approximation (CA) models, and robust optimisation (RO) models. IF models implicitly calculate the probability that a customer will be served by each facility (Snyder and Daskin 2005). RB models assume that each customer is backed up by a reliable facility under all disruption scenarios (Lim et al. 2010). In SB models facility failures are defined as explicit scenarios with their occurrence probabilities calculated based on the known probability of occurrence of local, regional or global disruption events (Azad and Hassini 2019; Aydin and Murat 2013). In CA models, customers are spread uniformly throughout some geographical area, and the parameters are expressed as a continuous function of the location Cui et al. (2010). When the uncertainty does not have a probabilistic description, robust optimisation is used in solving such problems (Cheng et al. 2021).

The literature review presented above reveals that no published study addresses the RCFLP when the failure probabilities are site-dependent. For this reason, in this paper, we present mathematical formulations and a Relax-and-Fix heuristic (RFH) to solve the given problem. RFH offers several advantages as a heuristic approach for solving combinatorial optimization problems (Absi and Heuvel 2019; Friske et al. 2022; Wang et al. 2022; Roshani et al. 2023) including location problems (Albareda-Sambola et al. 2013). The algorithm integrates two stages: fixing variables and relaxing constraints, providing a powerful framework for decomposing the problem into smaller, more manageable sub-problems (Joncour et al. 2023). Through iterative fixing of variables and relaxing of constraints, the algorithm efficiently explores the solution space, offering the potential for enhanced solution quality while minimizing computational effort (Giglio et al. 2017). To the best of our knowledge, the current research represents the first instance of utilizing a Fix-and-Relax heuristic for solving RCFLPs.

The main contributions of this paper are summarized in the following points:

- the best of our knowledge, this work is the first to tackle RCFLP when the failure probability of facilities is site-dependent;
- this paper presents a nonlinear mathematical formulation and its linear version for this problem for the first time;
- this paper is one of the first attempts to propose a Relax-and-Fix heuristic approach to solve RCFLPs.

The remainder of this paper is organized as follows. In Section 2, the problem is defined. Then, in Section 3, mathematical formulations for the considered problem are proposed. In Section 4, the solution approach is introduced. In Section 5, we discuss the performance of the proposed solution approach in solving experimental problem instances. Finally, conclusions and descriptions of future works will follow in section 6.

## Problem definition

The RCFLP problem addressed in this paper seeks to locate new facilities on a set of candidate locations to satisfy the demands of different existing customers in a two-echelon supply network. In this problem, it is assumed that customers’ demand is well forecasted and order quantities are known. The cost to ship a unit of demand from facility *j* to customer *i*, denoted by $d_{ij}$, is deterministic and known in advance. Facilities are at risk of failing due to disruptions caused by natural disasters, terrorist attacks, or labour strikes. It is assumed that the failure probability of facilities is site-dependent and their values are known. Associated with each facility *j* are the fixed location cost $f_{j}$ and the probability of failure $0< q_{j} < 1$. The events of facility disruptions are assumed to be independent.

Each facility has a specific throughput capacity to supply customers’ demands. Following Albareda-Sambola et al. (2017), it is assumed that the capacity limit of the facilities is strictly observed in the absence of any failures. Nevertheless, in the event of a disruption, operative facilities are allowed a slight exceeding of their capacities by $v_{j}$. Note that the maximum amount that facilities can exceed their normal capacity is specified.

Assuming that every customer’s demand must be met by one facility, we consider a cost ($\phi _{i}$) associated with each customer $i \in \mathcal {I}$. This cost represents the penalty incurred for not serving the customer per unit of missed demand and may be accrued even if some of the assigned facilities remain operational. This is under the condition that $\phi _{i}$ is less than the cost of serving *i* through any of these facilities. To model such a scenario, an "emergency" facility is introduced, denoted by $j = j_{0}$. The emergency facility has a fixed cost of $f_{j_{0}} = 0$, a failure probability of $q_{j_{0}} = 0$, and a transportation cost of $d_{ij_{0}} = \phi _{i}$ for the customer.

Facilities may experience failures, and to account for this, we use the level-r strategy proposed by Snyder and Daskin (2005). Specifically, we attempt to allocate the demand of a customer to different facilities at $r_{0}$ different levels. The customer’s demand is initially supplied by the facility assigned to it in the first level (level-0), and the other facilities in the subsequent levels serve as backup. In the event of a failure of the assigned facility in the primary level, the demand will be fulfilled by the facility to which the customer was assigned in the second level, and so on until the $r_{0}$th level. At optimality, every customer, denoted as *i* is expected to have precisely $r_{0}$ assignments unless she is assigned to the emergency facility at certain levels $s < r_{0}$. If customer *i* is allocated to exactly $r_{0}$ regular facilities spanning levels 0 through $r_{0}-1$, she must additionally be assigned to the emergency facility $j_{0}$ at level $r_{0}$. This accounts for the scenario where all $r_{0}$ regular facilities may potentially fail.

The objective is to minimize the total fixed costs of setting up facilities and the cost of transportation, in addition to the expected cost of disruptions, which includes the expected transportation and unsatisfied demands penalty costs.

**Table 1:** Numerical example

	Shipping costs					
	Candidate locations					
Customers	1	2	3	4	5	Demand
*a*	10	15	10	15	10	15
*b*	10	15	10	15	10	10
*c*	10	15	10	15	10	15
*d*	10	15	10	15	10	10
*e*	10	15	10	15	10	15
*f*	10	15	10	15	10	10
Capacity	20	20	20	20	20	
Failure probability	0.05	0.02	0.06	0.04	0.08	
Emergency cost	100	100	100	100	100	
Fixed location cost	1000	1000	1000	1000	1000	

To provide better insight into CRFLP and demonstrate the necessity of considering the site-dependent failure probability of facilities, a numerical example is presented in Table 1 and solved by applying the Mixed Integer Linear Programming formulation presented in Section 3.3. In this example, six customers with different demands should be served by facilities with same capacities, which can be located in five candidate sites. We assume that if a disruption occurs, up to three operational facilities can exceed at most 25 percent of their capacity to satisfy the demand of disrupted facilities.

This problem instance has also been solved by considering the failure probabilities of facilities identical and equal to 0.08. The generated solution for the problem is shown in solution 1 in Table 2. In this solution, facilities number 1, 2, 3, and 5 have been opened, and the total cost of the supply chain is 5723.6. Solution 2 in Table 2 illustrates the optimal solutions to this problem considering the actual site-dependent facilities’ failure probabilities. In this solution, facilities number 1, 3, 4, and 5 are opened to cover the demands of the customers. The total cost of the supply chain has been decreased to 5722.1. This example demonstrates how considering an identical failure probability for facilities may lead to finding non-optimal solutions for the CRFLP.

**Table 2:** The solution of the Numerical example

	Facilities											
	Solution 1 (*r*)						Solution 2 (*r*)					
Customers	0	1	2	3	4	5	0	1	2	3	4	5
*a*	2	5	3	1	6	-	4	1	3	5	6	-
*b*	3	5	1	2	6	-	3	1	5	4	6	-
*c*	5	3	1	2	6	-	1	5	3	4	6	-
*d*	3	1	5	2	6	-	6	-	-	-	-	-
*e*	6	-	-	-	-	-	5	1	3	4	6	-
*f*	1	3	5	2	6	-	3	5	1	4	6	-
-Opened facilities	{1,	2,	3,	5}			{1,	3,	4,	5}		
-Facilities experienced an		{1,	3}					{1,	3}			
increase in capacity												
-Expected Transportation Cost	723.6						722.1					
-Expected Penalty Cost	1000						1000					
-Fixed facility location Cost	4000						4000					
Total Costs	5723.6						5722.1					

## Mathematical formulation

After introducing the necessary notations, this section presents the mathematical formulation of the CRFLP. We employ both modelling techniques introduced by Cui et al. (2010) and Albareda-Sambola et al. (2017) in which they have applied the “level-*r*” assignment strategy to model their addressed RFLPs. A "level-*r*" assignment refers to a situation where there are *r* potentially failing facilities that are currently operational. When *r* equals 0, it is considered a primary assignment, otherwise, it is categorized as a backup assignment. For each customer *i*, there exists a level-*r* assignment for each *r* ranging from 0 to $j_{0}-1$, unless customer *i* is assigned to the non-failable facility ($j_{0}$). In simpler terms, customer *i* is assigned to one facility at level 0, another facility at level 1, and so on, until they have been assigned to all open facilities at a certain level or they have been assigned to the non-failable facility.

### Notations

#### Sets

- $\mathcal {I} = \{ 0, \ldots , {i_{0}-1} \}$ the set of customers;
- $\mathcal {F} = \{0, \ldots , j_0 - 1\}$: the set of failing facilities;
- $\mathcal{N}\mathcal{F} = \{j_0\}$: the set of non-failing facilities.
- $\mathcal {J} = {\mathcal {F} \cup \mathcal{N}\mathcal{F}}= \{ 0, \ldots , {j_{0}} \}$ the set of candidate facility locations;
- $\mathcal {R} = \{ 0, \ldots , {r_{0}} \}$ the set of levels;

#### Parameters

- $h_{i}$: demand of customer $i\in \mathcal {I}$;
- $d_{ij}$: the transportation cost per unit of demand served from $j\in \mathcal {J}$ to $i\in \mathcal {I}$;
- $c_{j}$: regular capacity of facility $j\in \mathcal {J}$;
- *v*: the limit on the expected demand exceeding each facility’s capacity;
- *b*: the limit on the number of facilities their expected demands exceed their capacity;
- $q_{j}$: probability of failure of failing facility $j\in \mathcal {F}$.

#### Decision variables

- $X_{j}\in \{0, 1\}$: binary facility location variable; $X_{j} = 1$ if facility $j\in \mathcal {J}$ is open; $X_{j} = 0$ otherwise;
- $Y_{ijr}\in \{0, 1\}$: binary assignment variable; $Y_{ijr} = 1$ if facility $j\in \mathcal {J}$ is assigned to customer $i\in \mathcal {I}$ at level $r\in \mathcal {R}$; $Y_{ijr} = 0$ otherwise;
- $P_{ijr}\ge 0$: probability that failing facility $j\in \mathcal {F}$ serves customer $i\in \mathcal {I}$ at level $r\in \mathcal {R}$;
- $U_{j}\in \{0, 1\}$: binary facility exceeding expected variable; $U_{j} = 1$ if the expected demand at facility $j\in \mathcal {J}$ exceeds its capacity $c_{j}$; $U_{j} = 0$ otherwise;
- $V_{j}\ge 0$: the amount by which the expected demand at facility $j\in \mathcal {J}$ exceeds its capacity;
- $$T_{c}$$: total cost;
- $C_{T}$: total expected transportation cost;
- $C_{L}$: total expected failure cost;
- $$C_{F}$$: fixed costs;

### Nonlinear formulations

Objective function:

1 $$\begin{aligned} \min T_{c} = C_{T}+C_{L}+C_{F} \end{aligned}$$

Constraints:

2 $$\begin{aligned} C_{T} = \sum _{i=0}^{{i_{0}}-1}\sum _{j=0}^{{j_{0}}-1}\sum _{r=0}^{{r_{0}}-1}h_{i}d_{ij}P_{ijr}Y_{ijr} \end{aligned}$$

3 $$\begin{aligned} C_{L} = \sum _{i=0}^{{i_{0}}-1}\sum _{r=0}^{{r_{0}}}h_{i}d_{ij_{0}}P_{i{j_{0}}r}Y_{i{j_{0}}r} \end{aligned}$$

4 $$\begin{aligned} C_{F} = \sum _{j=0}^{{j_{0}}-1}f_{j}X_{j} \end{aligned}$$

5 $$\begin{aligned} \sum _{j=0}^{{j_{0}}-1}Y_{ijr}+\sum _{s=0}^{r}Y_{i{j_{0}}s}= 1 , \quad 0\le i\le {i_{0}}-1, \quad 0\le r\le {r_{0}} \end{aligned}$$

6 $$\begin{aligned} \sum _{r=0}^{{r_{0}}-1}Y_{ijr} \le X_{j} \, \quad 0\le i\le {i_{0}}-1, \quad 0\le j\le {j_{0}}-1 \end{aligned}$$

7 $$\begin{aligned} \sum _{i=0}^{{i_{0}}-1}h_{i}Y_{ij0} \le c_{j}X_{j} \quad 0\le j\le {j_{0}}-1 \end{aligned}$$

8 $$\begin{aligned} \frac{1}{(1-q_{j})}\sum _{i=0}^{{i_{0}}-1}h_{i}\sum _{r=0}^{{r_{0}}}P_{ijr}Y_{ijr}\le c_{j}X_{j}+V_{j} \quad 0\le j\le {j_{0}} \end{aligned}$$

9 $$\begin{aligned} V_{j}\le vU_{j},\quad 0\le j\le {j_{0}}-1 \end{aligned}$$

10 $$\begin{aligned} \sum _{j=0}^{{j_{0}}-1}U_{j}\le b \end{aligned}$$

11 $$\begin{aligned} \sum _{r=0}^{{r_{0}}} Y_{i{j_{0}}r}=1, \quad 0\le i\le {i_{0}}-1 \end{aligned}$$

12 $$\begin{aligned} P_{ij0}=1-q_{j} , \, 0\le i\le {i_{0}}-1, \quad 0\le j\le {j_{0}} \end{aligned}$$

13 $$\begin{aligned} P_{ijr}=(1-q_{j})\sum _{k=0}^{{j_{0}}-1} \frac{q_{k}}{1-q_{k}}P_{ikr-1}Y_{ikr-1}, \quad 0\le i\le {i_{0}}-1, \quad 0\le j\le {j_{0}}, 1 \le r \le {r_{0}} \end{aligned}$$

The objective function (1) minimizes the total sum of expected transportation costs, expected failure costs, and fixed costs, whose values are calculated in constraints 2, 3, and 4, respectively. Constraints (5) state that every customer *i* and each level *r* must either be allocated to the emergency facility *J* (where *s* is less than *r*) or a regular facility at level *r*. Constraints (6) forbid the assignment of customers to a facility which is not open. Constraints (6) also implies that one customer must not be assigned to the same facilities at different levels. Constraints (7) are the capacity constraints in the scenario where no facility fails. Constraints (8)-(10) are modified versions of constraints proposed by Albareda-Sambola et al. (2017) introduced to limit the expected load of facilities overall disruption scenarios. More specifically, constraints (8) are utilized to calculate the values of variables $V_{j}$. On the other hand, constraint (9) serves a dual purpose of identifying the facilities where these variables have positive values and setting their maximum value, *v*. Constraint (10) imposes a restriction on the number of facilities where the $V_{j}$ variables can have positive values, limited by *b*. Constraints (11) enforce that every customer is assigned to an emergency facility at a specific level. Constraints (12) and (13) are the ”transitional probability” equations proposed by Cui et al. (2010) to calculate, $P_{ijr}$, the probability that facility *j* serves customer *i* at level *r*. Constraints (12) calculate the probability that facility *j* is open, given that $r=0$. For $r=1$ to $r={r_{0}}$, constraints (13) calculate $P_{ijr}$ as $(q_{k}(1-q_{j})/(1-q_{j}))P_{ik(r-1)}$, conditioned on the event that facility *k* serves customer *i* at level $r-1$. Note that constraints (lb) imply that $Y_{ik(r-1)}$ can equal 1 for at most one $k\in {j_{0}}$, which guarantees the correctness of the transitional probabilities (Cui et al. 2010).

### Linear formulations

The mathematical formulation is presented in subsection 3.2 because $P_{ijr}Y_{ijr}$, $0\le i\le {i_{0}}-1$, $0\le j\le {j_{0}}$, and $0\le r\le {r_{0}}$, is nonlinear. These nonlinear terms are a product of a continuous variable ($P_{ijr}$) and a binary variable ($Y_{ijr}$). As suggested by Cui et al. (2010) by applying the linearization technique introduced by Sherali and Alameddine (1992), we replace each $P_{ijr}Y_{ijr}$ with a new variable $W_{ijr}$ and add a set of new constraints to the formulation to enforce $W_{ijr}=P_{ijr}Y_{ijr}$ as follows:

14 $$\begin{aligned} W_{ijr}\le P_{ijr} \quad \forall i\in \mathcal {I}, \forall j\in \mathcal {J}, \forall r \in \mathcal {R} \end{aligned}$$

15 $$\begin{aligned} W_{ijr}\le Y_{ijr} \quad \forall i\in \mathcal {I}, \forall j\in \mathcal {J}, \forall r \in \mathcal {R} \end{aligned}$$

16 $$\begin{aligned} W_{ijr}\ge 0 \quad \forall i\in \mathcal {I}, \forall j\in \mathcal {J}, \forall r \in \mathcal {R} \end{aligned}$$

17 $$\begin{aligned} W_{ijr}\ge Y_{ijr}+P_{ijr}-1 \quad \forall i\in \mathcal {I}, \forall j\in \mathcal {J}, \forall r \in \mathcal {R} \end{aligned}$$

Considering the constraints mentioned above, the linearised formulation can be stated as:

(LRCFLP)

Objective function:

18 $$\begin{aligned} \min T_{c} = C_{T}+C_{L}+C_{F} \end{aligned}$$

Constraints:

19 $$\begin{aligned} C_{T} = \sum _{i=0}^{i_{0}-1}\sum _{j=0}^{j_{0}-1}\sum _{r=0}^{{r_{0}-1}}h_{i}d_{ij}W_{ijr} \end{aligned}$$

20 $$\begin{aligned} C_{L} = \sum _{i=0}^{I-1}\sum _{r=0}^{{r_{0}}}h_{i}d_{iJ}W_{i{j_{0}}r} \end{aligned}$$

$$\begin{aligned} (4)-(7) \end{aligned}$$

21 $$\begin{aligned} \frac{1}{(1-q_{j})}\sum _{i=1}^{I-1}h_{i}\sum _{r=1}^{{r_{0}}-1}W_{ijr}\le c_{j}X_{j}+V_{j} \quad 0\le j\le {j_{0}}-1 \end{aligned}$$

$$\begin{aligned} (9)-(12) \end{aligned}$$

22 $$\begin{aligned} P_{ijr}=(1-q_{j})\sum _{k\in F} \frac{q_{k}}{1-q_{k}}W_{ikr-1}, \quad 0\le i\le {i_{0}}-1, \quad 0\le j\le {j_{0}}, \quad 1 \le r \le {r_{0}} \end{aligned}$$

$$\begin{aligned} (14)-(17) \end{aligned}$$

In our formulation, we do not require that a customer be served by the nearest open facility, which contrasts with the approach commonly taken in classic facility location problems. Snyder and Daskin (2005) and Cui et al. (2010) demonstrated that the optimal solution for the RUFLP exhibits a consistent pattern of assigning customers to open facilities. When all facilities have identical failure probabilities, the optimal solution assigns customers sequentially in increasing order of distance. Remarkably, this pattern persists even when failure probabilities vary across facility sites. However, the following proposition demonstrates that this result does not hold in the RCFLP when facility failure probabilities differ across sites.

### Remark 1

In any optimal solution of LRCFLP, if $Y_{ijr}=1$ and $Y_{ik,r+1}=1$, then $d_{ij}$ is not necessarily less than or equal $d_{ik}$.

Proposition 1 indicates that the optimal levels of assignment for a specific group of facilities allocated to a customer are not determined solely by the distances between the customer and those facilities. This means that, as demonstrated by the example that follows, it may be possible to assign a customer to a facility that is farther away and still achieve an optimal solution This is because, in RCFLPs, the capacity constraints of the facilities can sometimes prevent the assignment of customer orders to them according to the rule that exists in the optimal solution of RUFLPs.

### Example 1

Consider the numerical example presented in Section 2. Table 2 shows the assignment of facilities to customers across five different levels. Consider customer *e*. This customer is assigned to facility number 4 at level 0 and to facility number 1 at a higher, level 1. However, the distance from facility 4 to customer *e* is greater than the distance from the given customer to facility 1.

## Proposed algorithm

The mathematical formulation presented in subsection 3.3 can be utilised to solve small-sized problem instances using commercial solvers such as Cplex and Gurobi. However, when the size of the problem grows, the computational time required to solve this problem increases exponentially. In this paper, a Relax-and-Fix MIP-based algorithm is proposed to solve the large-sized problem instances. The different components of the proposed relax-and-fix heuristic are explained in the following subsections.

### Proposed Relax-and-Fixed heuristic

Relax-and-fix heuristic (in the following denoted as RFH) is a technique used in integer programming to solve mixed-integer linear programming (MILP) problems (Giglio et al. 2017). The basic idea is to fix some variables to their current integer values and relax the other variables to be fractional. The relaxed problem can then be solved using standard linear programming techniques. After obtaining a solution, the fractional variables are rounded to the nearest integer, and the resulting solution is tested for feasibility (Silva et al. 2021; Roshani et al. 2023).

#### General outline

The proposed RFH solves the RCFLP problem in two phases: beginning, and ending. In each phase, RFH solves a partially relaxed or fixed version of the original model by applying one of the proposed relaxing and fixing (RF) strategies in subsection 4.1.2. Using RF strategies, the algorithm decides which binary variables should be fixed and remain binary, or will be relaxed in the partially relaxed and/or fixed models in each phase. In the beginning phase, a portion of the original model’s binary variables are selected using RF strategies and relaxed to become continuous variables, taking values from the interval [0, 1]. The remaining binary variable group is kept in the model as binary. In the ending stage, the value of some of the binary variables is "frozen," meaning they are fixed to the optimal values obtained by solving the partially relaxed model from the beginning phase. The remaining binary variables in the main model, defined as continuous in the beginning phase, are again considered binary.

#### Relaxing and fixing strategy

The RFH method uses relaxing and fixing strategies to make it easier to solve the problem in each iteration and reduce the computational burden. However, increasing the number of relaxed variables may simplify the problem but it can also lower the quality of the solutions. Therefore, it is crucial to develop appropriate relaxing and fixing strategies to create an efficient heuristic. In section 2, the RCFLP is described, which includes three sets of binary variables: location ($X_{j}$), assignment ($Y_{ijr}$), and facilities’ capacity exceeding indicator ($U_{j}$) variables. The location variables total $j_{0}$, the assignment variables total $i_{0}\cdot j_{0}\cdot r_{0}$, and the capacity exceeding variables total $j_{0}$.

The following relaxing and fixing strategies are presented: **Strategy 1:**

- **Beginning phase:**
  1. Let the location variables, $X_{j}, j=0,\cdots ,{j_{0}}$, be binary;
  2. Let assignment variables for level 0 to level $\alpha$, ($Y_{ijr}, i=0,\cdots ,{i_{0}-1}, j=0,\cdots ,{j_{0}}, r=0,\cdots ,\alpha$), be binary, continuously relax all other assignment variables, ($Y_{ijr}, i=1,\cdots ,{i_{0}}, j=1,\cdots ,j_{0}, r=\alpha +1,\cdots ,{r_{0}}$), allowing values between zero and one;
  3. Continuously relax all facilities’ capacity exceeding indicator variables ($U_{j}, j=1,\cdots ,{j_{0}}$) allowing values between zero and one.
- **Ending phase:**
  1. Fix the value of the location variables to the optimal values obtained in the beginning section;
  2. Set the value of assignment variables for those facilities that have not been opened to zero. Set the maximum number of assignment levels to the *number of opened facilities +1*. Let the remaining assignment variables be binary;
  3. Fix the value of capacity exceeding indicator variables to one if the value of their optimal solution obtained in the beginning section is one; Let the rest of capacity exceeding indicator variables be binary.

**Strategy 2:**

- **Beginning phase:**
  1. Let the location variables, $X_{j}, j=0,\cdots ,{j_{0}}$, be binary;
  2. Let assignment variables for level 0 to level $\alpha$, ($Y_{ijr}, i=0,\cdots ,i_{0}-1, j=0,\cdots ,{j_{0}}, r=0,\cdots ,\alpha$), be binary, set all other assignment variables, ($Y_{ijr}, i=1,\cdots ,{i_{0}}, j=1,\cdots ,{j_{0}}, r=\alpha +1,\cdots ,{r_{0}}$), to zero;
  3. Continuously relax all facilities’ capacity exceeding indicator variables ($U_{j}, j=1,\cdots ,{j_{0}}$) allowing values between zero and one.
- **Ending phase:** The steps are similar to the ending phase of strategy 1.

The aim of the initial phase in the mentioned strategies is to determine which facilities should be opened. In other words, this phase of the algorithm calculates the value of the location variables, $X_{j}, j=1,\cdots ,{j_{0}}-1$. The solution generated by the model in the initial phase will then be used to reduce the number of assignment binary variables in two ways. First, in the ending phase, the value of assignment variables related to those facilities that have not been opened is set to zero. Second, the value of R, the number of levels, is limited to the number of opened facilities.

The number of binary variables in the partially relaxed model generated by proposed RFH in the beginning phase when it applies both **Strategy 1**, and **Strategy 2** is equal to $J + (I * J*(\alpha +1))$. Furthermore, the number of binary variables in the partially fixed model generated in the ending phase depends on the number of opened facilities and the number of facilities whose related capacity exceeding variables ($U_{j}$) value equals one in the solution of the model generated in the beginning phase. Suppose that $N_{1}$ out of ${j_{0}}$ facilities has not been opened, and the value of capacity exceeding indicator variables for $N_{2}$ out of ${j_{0}}$ facilities equals one in the optimal solution of the model generated in the beginning phase. Then, the number of binary variables in the model generated in the ending phase is $(({j_{0}}-N_{2}) + i_{0} * ({j_{0}} - N_{1}) * ({r_{0}} - N_{1}))$. The more facilities that are opened the fewer facilities exceed their capacity, and the more complex the partially fixed problem in the ending phase becomes. Some preliminary experiments show that the proposed RFH, using strategy 1 will take longer to solve problem instances compared to using strategy 2. The reason for this is that the partially relaxed model generated in the first phase of strategy 1 includes several continuously relaxed variables whose values are fixed to zero in the partially relaxed and fixed model generated in the initial phase of strategy 2. However, because the model generated by Strategy 1 includes relaxed allocation variables, it has the potential to produce a better approximation of the solution. Therefore, in the presented method, we utilize both strategies.

### Numerical Example

**Fig. 1:**
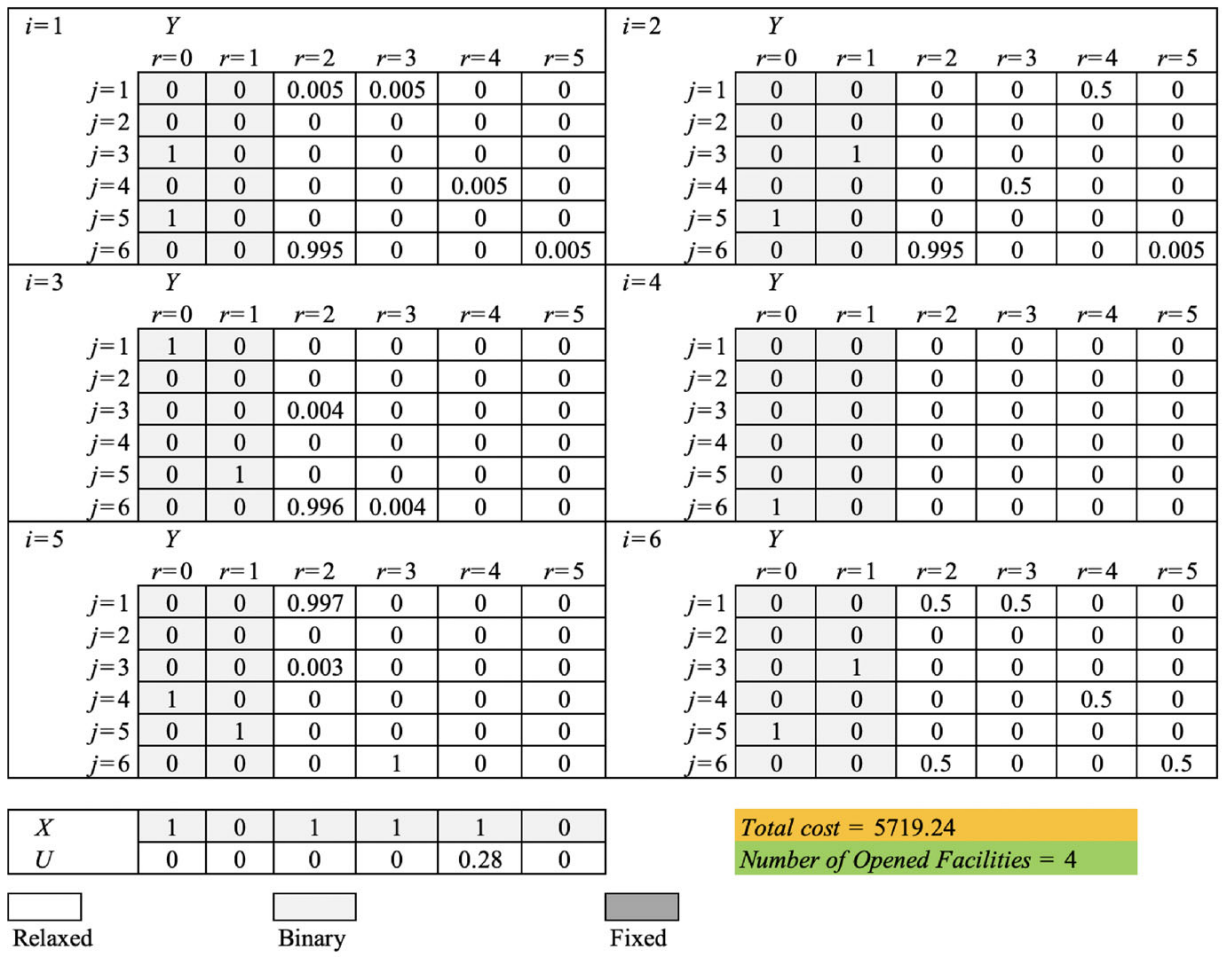
The value of binary variables of the original model found by RFH for the example instance in the beginning section

**Fig. 2:**
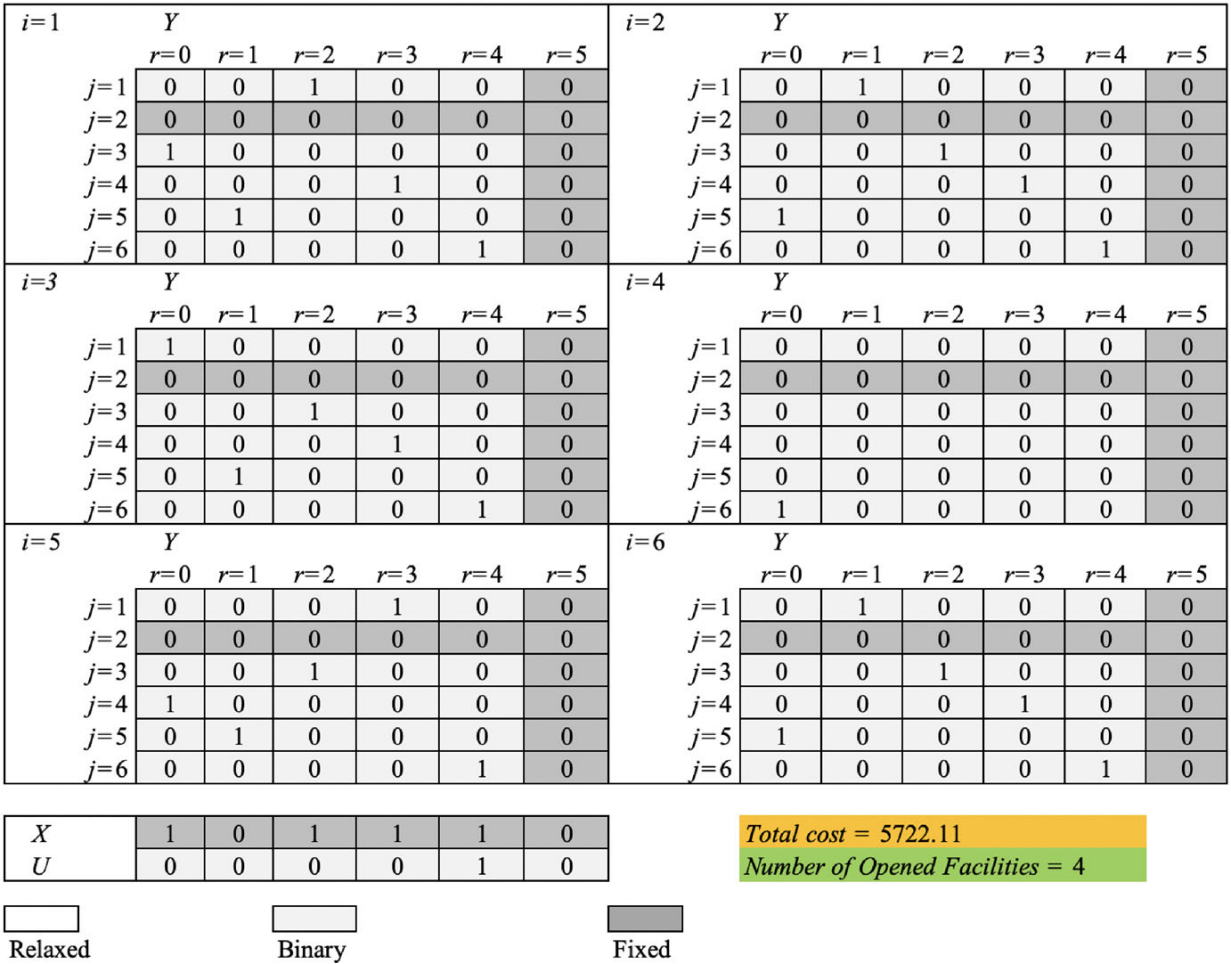
The value of binary variables of the original model found by RFH for the example instance in the ending section

The RFH with relaxing and fixing strategy proposed in subsection 4.1.2 is here applied to solve the numerical example presented in subsection 4.1.2. The proposed heuristic was coded in a Java environment using IBM ILOG Cplex 22.1 Concert Technology. The instance was solved by considering the 2 levels of the assignment variables in the beginning section by setting $\alpha = 1$. Figures 1 and 2 show the process of fixing and relaxing the binary variables of the original model by RFH for this numerical example in the beginning and ending sections, respectively.

In the beginning stage, the location and assignment binary variables of levels 0 and 1 are considered, and the assignment variables are relaxed for the other levels. In addition, the capacity exceeding indicator variables are relaxed too. Figure 1 shows the process of fixing and relaxing the binary variables by RFH for this numerical example in the beginning section. The value of the objective function after solving the partially relaxed problem generated in the beginning stage is 5719.24 and the number of opened facilities is 4. In the ending stage, the binary location variables and those capacity-exceeding variables whose values are previously set to one in the solution of the partially relaxed model are fixed at their optimal values identified during the beginning stage. The assignment variables and facilities’ capacity exceeding indicator variables whose values are less than one in the optimal solution of the model previously generated are set as binaries. After solving this model, the final feasible solution found by the algorithm is reported with the objective function equal to 5722.11. The optimal solution of the partially fixed model, generated in the ending section by the proposed RFH, is shown in Figure 2. Comparing the results generated by RFH and the value of the optimal objective function reported in Solution 2 in Table 2 indicates that RFH has been able to generate a solution with only a 0.01 deviation from the value of the optimal objective function (i.e. 5722.1).

Table 3 shows the results of the proposed algorithm in solving the problem by considering different values of $\alpha$ for both proposed relax-and-fix strategies. As observed, when the proposed algorithm uses strategy 1, the obtained values for the model generated in the beginning phase represent a lower value for the value of the objective function of the generated model in the ending phase (i.e. 5722.11). However, by using strategy 2, the objective function values of the model generated in phase 1 are greater or equal to the objective function values of the model generated in phase 2 and the optimal value for the objective function of the original model.

**Table 3:** The values of objective functions of the models generated in phases 1 and 2 by proposed RFH when using both relax-and-fix strategies

	Strategy 1			Strategy 2		
$\alpha$	Phase 1	Phase 2	Opened facilities	Phase 1	Phase 2	Opened facilities
1	5719.24	5722.11	1, 3, 4, 5	6029.5	5723.59	1, 2, 3, 5
2	5721.85	5722.11	1, 3, 4, 5	5739.55	5722.11	1, 3, 4, 5
3	5722.10	5722.11	1, 3, 4, 5	5723.24	5722.11	1, 3, 4, 5
4	5722.11	5722.11	1, 3, 4, 5	5722.11	5722.11	1, 3, 4, 5

## Experimental results and discussion

In this section, we test the performance of the proposed RFH algorithm on a number of experimental instances collected from the literature and modified based on the characteristics of RCFLPs. First, in subsection 5.1 the test problems and the value of the RFH algorithm’s parameters are introduced. Second, in subsection 5.2, the performance of the proposed RFH algorithm will be compared with the CPLEX commercial solver used to solve the proposed linear mixed-integer formulation LRCFLP. Third, in subsection 5.4 the sensitivity analysis of failure probabilities on the results is presented.

### Data set and parameter settings

To test the performance of the algorithm, three datasets related to capacitated P-median problem instances with 49, 50, 88, and 150 nodes have been selected^1^ and edited based on the characteristics of CRFLPs. In particular, 46 experimental instances were generated with different levels of *b* and *v* parameters. In all instances, *v* parameter is set to $a \times c[j]$, where *a* is the percentage of the capacity of facility *j* that can be added to its capacity in case of failure. Table 4 summarizes the values of the parameters used in generating experimental instances.

The failure probability of facilities is set to a randomly generated number from a (0, 0.1) interval. The capacity level of the facilities is considered to be very tight and set to 500, 1000, 800, and 1000 in the problem instances with 49, 50, 88, and 100 nodes, respectively. To evaluate the performance of the proposed RFH, we solved this set of problem instances by implementing the proposed linear model (as detailed in subsection 3.3) using CPLEX, while the RFH algorithm was implemented in a Java environment. Additionally, we utilized CPLEX Concert technology to solve the partially relaxed and fixed models. To ensure a fair comparison, both CPLEX and RFH were executed under two CPU time limits: 800 and 1600 seconds, respectively. Subsequently, the results were compared.

The performance of the proposed RFH, in terms of solution quality and computational time, is influenced by three parameters: $\alpha$, the maximum elapsed time (timelimit), and the MIP solver tolerance for the optimality gap (OptGap). Some preliminary experiments were conducted to determine the most appropriate configuration of these parameters. Table 5 summarizes the values of the RFH parameters. In this table, two different values for the time limit are provided. The first value represents the time limit for the experimental studies with the entire algorithm set to 800 seconds, while the second one is associated with a set of experimental studies using a time limit of 1600 seconds for the entire RFH.

**Table 4:** The value of parameters for generating experimental instances

Nodes	$\mathcal {I}$	$\mathcal {J}$	$\mathcal {F}$	$\mathcal{N}\mathcal{F}$	*b*	*a*	$c_{j}$
49	$\{0,\cdots , 48\}$	$\{0,\cdots , 49\}$	$\{0,\cdots , 48\}$	$\{49\}$	0, 3, 5, 7	0, 0.05, 0.10, 0.15	500
50	$\{0,\cdots , 49\}$	$\{0,\cdots , 50\}$	$\{0,\cdots , 49\}$	$\{50\}$	0, 3, 5, 10	0, 0.05, 0.10, 0.15	1000
88	$\{0,\cdots , 87\}$	$\{0,\cdots , 88\}$	$\{0,\cdots , 87\}$	$\{88\}$	0, 3, 5, 7, 10	0, 0.05, 0.10, 0.15	800
150	$\{0,\cdots , 149\}$	$\{0,\cdots , 150\}$	$\{0,\cdots , 149\}$	$\{150\}$	0, 3, 5, 7, 10	0, 0.05, 0.10, 0.15	1000

**Table 5:** The value of parameters of the RFH algorithm

		Timelimit		OptGap	
Nodes	$\alpha$	Phase 1	Phase 2	Phase 1	Phase 2
49	$\lfloor \frac{49}{4}\rfloor$	600 & 1300	200 & 300	0.0001	0.001
50	$\lfloor \frac{50}{4}\rfloor$	400 & 400	800 & 800	0.0001	0.001
88	$\lfloor \frac{88}{4}\rfloor$	600 & 1300	200 & 300	0.0001	0.001
150	$\lfloor \frac{150}{50}\rfloor$	600 & 1200	200 & 400	0.0001	0.001

### Performance of the proposed algorithm

In this subsection, the tests to evaluate the performance of the RFH on the set of experimental instances introduced in subsection 5.1 are presented. The proposed heuristic was coded in a Java environment and for RFH the partially relaxed models were solved using IBM ILOG CPLEX 22.1 Concert Technology. The results of RFH are compared with the MILP formulation results proposed in Section 3.3 which was coded in IBM ILOG CPLEX 22.1. All the experiments were performed on a personal computer with Apple M1, 8 Cores CPU, and 8 GB RAM. Table 6 presents the results obtained.

In table 6, the specifications of instances are in the first four columns: the number of nodes, time limits, the limit on the number of facilities their expected demands exceed their capacity (*b*), and the limit on the expected demand exceeding each facility’s capacity multiplier (*a*). The next five columns in Table 6 report the results found by the CPLEX for the original MILP model, showing the number of opened facilities ($\#$), the lower bound (LB), the upper bound (UB) of the objective function, the optimality gap (Gap) between such two bounds (computed as $\frac{UB-LB}{UB}$), as well as the Cpu time (in seconds). The next four columns in these tables include the results found by the proposed RFH, in particular, the number of opened facilities ($\#$), the upper bound (UB) of the objective function, the optimality gap (Gap) between such two bounds, as well as the Cpu time (in seconds). To evaluate the quality of the solutions found by RFH, table 6 reports in the last two columns two improvement indices, namely $Imp_{1}$, and $Imp_{2}$. $Imp_{1}$ is the percentage improvement of the best cost produced by RFH from the solution found by the MIP solver (i.e., UB); it is computed as $100\cdot \frac{UB_{MIP}-UB_{RFH}}{UB_{RFH}}$. $Imp_{2}$ is the percentage improvement of the MIP gap from the RFH gap; it is given by $100\cdot \frac{Gap_{MIP}-Gap_{RFH}}{Gap_{RFH}}$. Note that values of $Imp_{1}$, and $Imp_{2}$ less than zero denote that RFH produces worse cost, and a worse gap than the MIP solver, respectively. In addition, for each set of experimental instances with same number of nodes, the average value obtained for each column is presented based on the algorithm’s execution time limit in *Ave* rows.

**Table 6:** Computational results of MIP solver, and the RFH algorithm for the test instances

Node	Timelimit	b	*a*	MIP					RFH					
				#	*LB*	*UB*	Gap	Cpu(s)	#	*UB*	Gap	Cpu(s)	$Imp_{1}$	$Imp_{2}$
49	800	0	0	11	1200046.2	1258960.31	4.68	812.1	10	1246963.37	3.76	662.4	0.96	24.38
		3	0.05	8	1141179.64	1175356.01	2.91	812.2	8	1173639.37	2.77	624.5	0.15	5.216
			0.1	8	1149160.92	1174599.36	2.16	808.5	8	1173624.75	2.08	624.6	0.08	3.62
			0.15	8	1143187.49	1173706.81	2.6	807.8	8	1173665.5	2.6	622.4	0	0.12
		5	0.05	8	1141537.37	1174675.86	2.82	808.1	8	1174719.25	2.82	621.9	-0	-0.17
			0.1	8	1141928.82	1174818.75	2.8	807.6	8	1173670.75	2.7	624.7	0.1	3.53
			0.15	8	1142511.75	1174991.78	2.76	808.1	8	1173665.5	2.65	622.2	0.11	3.98
		7	0.05	8	1140394.6	1175161.04	2.96	818.3	8	1174719.25	2.92	613.8	0.04	1.30
			0.1	7	1122268.28	1212795.1	7.46	1163.7	8	1174459.87	4.44	630.5	3.26	67.87
			0.15	7	1122268.28	1212795.1	7.46	2008.5	8	1174459.87	4.44	630.5	3.26	67.87
	Ave			8.1	1144448.33	1190786.01	3.86	965.49	8.2	1181358.75	3.12	627.75	0.8	17.77
	1600	0	0	11	1205703.97	1258843.54	4.22	1609.7	10	1246794.75	3.3	1607.8	0.97	28.05
		3	0.05	8	1144520.54	1174616.95	2.56	1600	8	1173573.5	2.48	1328.6	0.09	3.409
			0.1	8	1151211.66	1174604.78	1.99	1600	8	1173573.5	1.91	1327	0.09	4.437
			0.15	8	1145806.81	1174599	2.45	1600	8	1173573.62	2.37	1323.5	0.09	3.55
		5	0.05	8	1140661.38	1175422.73	2.95	1600	8	1173573.5	2.8	1329.3	0.16	5.19
			0.1	8	1144647.87	1174626.38	2.55	1600	8	1173573.5	2.46	1326	0.09	3.459
			0.15	8	1145436.72	1174608.26	2.48	1600	8	1173573.62	2.4	1324.5	0.09	3.439
		7	0.05	8	1145018.15	1174657.49	2.523	1604.4	8	1174598	2.52	1605.4	0.01	0.187
			0.1	8	1144531.28	1174652.5	2.56	1609.1	8	1174598	2.56	1326.8	0	0.01
			0.15	8	1145705.07	1174603.02	2.46	1608.4	8	1173573.5	2.37	1323.8	0.09	3.594
	Ave			8.3	1151324.345	1183123.465	2.6743	1603.16	8.2	1181100.549	2.52	1382.3	0.17	5.532
50	800	0	0	46	56003.58	56871.67	1.53	809.6	46	56802.72	1.41	807.9	0.12	8.752
		3	0.05	46	55403.17	56721.39	2.32	809.5	46	56715.88	2.31	808.1	0.01	0.236
			0.1	46	55229.51	56756.71	2.69	809.1	46	56795.17	2.76	806.7	-0.1	-2.42
			0.15	46	55320.87	56743.64	2.51	810.3	46	56878.1	2.74	806.7	-0.2	-8.32
		5	0.05	46	55311.72	56738.91	2.51	809.7	46	56719.24	2.32	808.8	0	-0.01
			0.1	46	54160.57	56787.98	4.63	810.5	46	56725.44	2.64	808.3	0.06	2.004
			0.15	46	55443.38	56714.66	2.24	809.7	46	56717.48	2.46	807.52	0.05	1.933
		10	0.05	46	55606.08	56676.75	1.89	809.4	46	56691.43	2.43	807.3	0.08	3.134
			0.1	46	54160.6	56820.92	4.68	810.1	46	56784.06	4.62	807.7	0.01	0.214
			0.15	46	55421.02	56712.34	2.27	809.6	46	56673.13	2.17	806.8	0.07	3.231
	Ave			46	55206.05	56754.497	2.727	809.75	46	56750.265	2.59	807.58	0.01	0.875
	1600	0	0	46	56031.87	56659.07	1.11	1600	46	56643.83	1.08	1610.9	0.03	2.743
		3	0.05	46	55454.68	56651.93	2.11	1600	46	56700.05	2.2	1609.9	-0.1	-3.93
			0.1	46	55386.58	56685.8	2.29	1600	46	56709.72	2.33	1607.6	-0	-1.85
			0.15	46	55411.04	56657.94	2.2	1600	46	56725.24	2.32	1608.2	-0.1	-5.04
		5	0.05	46	55325.26	56670.09	2.37	1600	46	56648.86	2.34	1609.5	0.04	1.434
			0.1	46	54194.36	56675.3	4.38	1600	46	56647.87	4.33	1609.8	0.05	1.128
			0.15	46	55535.37	56658.54	1.98	1600	46	56638.89	1.95	1607.6	0.03	1.625
		10	0.05	46	56141.02	56665.94	0.9	1600	46	56660.4	0.92	1608.5	0.01	-1.82
			0.1	46	54216.51	56713.02	4.4	1600	46	56639.64	4.28	1608.7	0.13	2.848
			0.15	46	55521.29	56658.81	2.01	1600	46	56797.66	2.25	1609.7	-0.2	-10.6
	Ave			46	55321.798	56669.644	2.375	1600	46	56681.216	2.4	1609	-0	-1.34
88	800	0	0	12	1599804.08	1802793.05	11.26	868.8	12	1710814.25	6.49	729.4	5.38	73.53
		3	0.05	12	1600370.4	1836418.9	12.85	866.1	12	1710219.37	6.42	721.2	7.38	100.1
			0.1	12	1600441.4	1836170.21	12.83	872.6	12	1710190.37	6.42	719	7.37	99.93
			0.15	12	1600108.7	1807689.91	11.48	864.4	12	1710252.62	6.44	728.8	5.7	78.25
		5	0.05	12	1600370.4	1836418.9	12.85	864.6	12	1710219.37	6.42	717.8	7.38	100.1
			0.1	12	1600158.84	1835177.55	12.81	863.3	12	1710190.37	6.43	719	7.31	99.1
			0.15	12	1600108.7	1807689.91	11.48	863.5	12	1710225.62	6.44	723.5	5.7	78.3
		7	0.05	12	1600370.4	1836418.9	12.85	864.6	12	1720339.87	6.97	730.5	6.75	84.27
			0.1	12	1600570.03	1835177.55	12.78	872.8	12	1710235.25	6.41	723.8	7.31	99.3
			0.15	12	1600353.15	1807689.91	11.47	863.2	12	1710261.25	6.43	724.3	5.7	78.48
		10	0.05	12	1600475.38	1836418.9	12.85	865.8	12	1710219.37	6.42	714.8	7.38	100.3
			0.1	12	1600371.93	1835177.55	12.79	867.9	12	1710240.6	6.42	719.6	7.31	99.09
			0.15	12	1600353.15	1807689.91	11.47	866.3	12	1710261.25	6.43	722.4	5.7	78.48
	Ave			12	1600296.66	1824687.012	12.29	866.45	12	1711051.505	6.47	722.62	6.64	89.93
	1600	0	0	12	1604989.1	1802793.05	10.97	1700	12	1710686.87	6.18	1657.4	5.38	77.55
		3	0.05	12	1606068.45	1836418.9	12.54	1700	12	1710162.25	6.09	1637.3	7.38	106
			0.1	12	1606042.54	1836170.21	12.53	1700	12	1710162.25	6.09	1640.8	7.37	105.8
			0.15	12	1605224.12	1766019.84	9.1	1700	12	1710163.12	6.14	1349.4	3.27	48.3
		5	0.05	12	1606068.45	1836418.9	12.54	1700	12	1710162.5	6.09	1639.1	7.38	106
			0.1	12	1605798.94	1835177.55	12.5	1700	12	1710162.25	6.1	1654.5	7.31	104.8
			0.15	12	1605252.9	1766019.84	9.1	1700	12	1710163.12	6.13	1349.3	3.27	48.34
		7	0.05	12	1606068.45	1767449.65	9.13	1700	12	1710162.5	6.09	1638.7	3.35	50
			0.1	12	1605798.94	1835177.55	12.49	1700	12	1710162.12	6.1	1638.6	7.31	104.7
			0.15	12	1605252.9	1766019.84	9.1	1700	12	1710163.12	6.13	1353.3	3.27	48.34
		10	0.05	12	1606068.45	1836418.9	12.54	1700	12	1710162.37	6.09	1640.5	7.38	106
			0.1	12	1605586.33	1835177.55	12.51	1700	12	1710162.12	6.11	1643.5	7.31	104.6
			0.15	12	1605224.12	1807689.91	11.2	1700	12	1710163.12	6.14	1351.6	5.7	82.52
	Ave			12	1605649.51	1809765.51	11.25	1700	12	1710202.90	6.11	1553.4	5.82	84.08
150	800	0	0	-	-	-	-	-	25	3348183.75	NA	890.9	NA	NA
		3	0.05	-	-	-	-	-	17	2725520.25	NA	888.2	NA	NA
			0.1	-	-	-	-	-	20	2900598.5	NA	878.5	NA	NA
			0.15	-	-	-	-	-	15	2529450.25	NA	879.3	NA	NA
		5	0.05	-	-	-	-	-	17	2725812.25	NA	878.1	NA	NA
			0.1	-	-	-	-	-	20	2900437	NA	878.4	NA	NA
			0.15	-	-	-	-	-	15	2529042.75	NA	874.9	NA	NA
		7	0.05	-	-	-	-	-	17	2724743.5	NA	882.1	NA	NA
			0.1	-	-	-	-	-	20	2900598.5	NA	877.6	NA	NA
			0.15	-	-	-	-	-	15	2529450.25	NA	874.2	NA	NA
		10	0.05	-	-	-	-	-	17	2724830	NA	878.1	NA	NA
			0.1	-	-	-	-	-	20	2901239.25	NA	883.9	NA	NA
			0.15	-	-	-	-	-	15	2529322.25	NA	880.2	NA	NA
	Ave			-	-	-	-	-	17.92	2766863.73	NA	880.33	NA	NA
	1600	0	0	-	-	-	-	-	12	2414217.75	NA	1702.5	NA	NA
		3	0.05	-	-	-	-	-	11	2362812.5	NA	1703.6	NA	NA
			0.1	-	-	-	-	-	14	2466310.5	NA	1724.5	NA	NA
			0.15	-	-	-	-	-	12	2362431	NA	1707.3	NA	NA
		5	0.05	-	-	-	-	-	12	2355155.75	NA	1701.6	NA	NA
			0.1	-	-	-	-	-	14	2466378.5	NA	1704.8	NA	NA
			0.15	-	-	-	-	-	12	2362429	NA	1706.7	NA	NA
		7	0.05	-	-	-	-	-	11	2363040.5	NA	1699.8	NA	NA
			0.1	-	-	-	-	-	14	2466344.75	NA	1704.3	NA	NA
			0.15	-	-	-	-	-	12	2362433.75	NA	1699.2	NA	NA
		10	0.05	-	-	-	-	-	12	2355373.5	NA	1706.4	NA	NA
			0.1	-	-	-	-	-	14	2466311.25	NA	1711.3	NA	NA
			0.15	-	-	-	-	-	12	2362432.5	NA	1700.3	NA	NA
	Ave			-	-	-	-	-	12.46154	2397359.33	NA	1705.56	NA	NA

After reviewing the results presented in Table 6, it can be concluded that RFH outperformed CPLEX in nine out of ten cases when the time limit was set to 800 seconds, for problem instances with 49 nodes. Only in one problem instance ($b=5$, $a=0.05$) did RFH produce a solution that was slightly inferior to CPLEX, with $Imp_{2}$ of -0.16%. However, when the time limit was increased to 1600, RFH consistently produced better results across all 49-node instances, achieving average values of 0.17 and 5.53 for $Imp_{1}$ and $Imp_{2}$, respectively.

In the 50-node experimental instances, when the time limit was set to 800 seconds, RFH generated better solutions than CPLEX in seven instances. However, in five instances, RFH produced slightly worse solutions. When the time limit was increased to 1600 seconds, RFH performed better in five out of the 12 problem instances, with average values of 0 and -1.34 for $Imp_{1}$ and $Imp_{2}$, respectively, across all the experimental instances in this set.

The proposed algorithm has been proven to be more effective than CPLEX in solving larger problems that involve 88 and 150 nodes. In both sets of problems, the proposed algorithm consistently found better solutions. On average, the values of imp1 and imp2 for problems with 88 nodes are 6.64% and 89.93%, respectively, when the time limit is set to 800 seconds. For the same set of problems, the values are 5.82% and 84.08% when the time limit is set to 1600 seconds. Regarding the problem with 150 nodes, the solution process has not been initiated by CPLEX because of the size of the problem. However, the proposed RFH has displayed the capability of generating solutions for these complicated problem instances. It should be noted that for this particular type of problem, the lower limit was not provided by CPLEX. Therefore, the optimality gap for the generated solution by RFH and the values of $Imp_{1}$ and $Imp_{2}$ could not be calculated (NA). Given these considerations and the complexity of the problem, it is reasonable to conclude that the RFH algorithm achieved satisfactory results for the test instances.

### The analysis of the expected capacity

In this section, we present the sensitivity analysis of facility capacity after failure. The solutions generated by the proposed algorithm within 1600 seconds are summarized in Table 7. The table includes not only the total cost of the supply chain ($T_{c}$) but also the breakdown of costs into transportation ($C_{T}$), penalty costs ($C_{L}$), facility fixed costs ($C_{F}$), and algorithm CPU time. Additionally, in each column, the lowest cost for problems with the same number of nodes is marked in bold.

**Table 7:** Computational results of the RFH algorithm for RCFLPs (capacity analysis)

Node	b	*v*	RCFLP					
			#	$C_{T}$	$C_{L}$	$C_{F}$	$T_{c}$	Cpu(s)
	0	0	10	**544312.13**	51.28	702600	1246963.37	1607.8
	3	0.05	8	612232.1	7.31	**561400**	1173639.37	1328.6
		0.1	8	612224.75	**0.19**	**561400**	1173624.75	1326.95
		0.15	8	612243.5	21.99	**561400**	1173665.5	1323.5
	5	0.05	8	608117.3	1.9	566600	1174719.25	1329.3
		0.1	8	612186.09	84.62	**561400**	1173670.75	1326
		0.15	8	612243.5	21.99	**561400**	1173665.5	1324.5
	7	0.05	8	608117.3	1.9	566600	1174719.25	1605.4
		0.1	8	608049.5	10.36	566600	1174659.87	1326.8
		0.15	8	612204.12	8.23	**561400**	**1173612.37**	1323.8
Ave			8.2	604193.03	20.98	577080	1181294	1382.26
50	0	0	46	7019.47	2990.24	**46793**	56802.72	1610.9
	3	0.05	46	7028.69	2894.19	**46793**	56715.88	1609.9
		0.1	46	6720.01	3282.16	**46793**	56795.17	1607.6
		0.15	46	7013.31	3072	**46793**	56878.1	1608.2
	5	0.05	46	6934.47	2991.77	**46793**	56719.24	1609.5
		0.1	46	**6473.1**	3459.33	**46793**	56725.43	1609.8
		0.15	46	6945.56	2978.91	**46793**	56717.48	1607.6
	10	0.05	46	6954.19	2944.24	**46793**	56691.43	1608.5
		0.1	46	7154.88	**2836.18**	**46793**	56784.06	1608.7
		0.15	46	6834.13	3046	**46793**	**56673.13**	1609.7
Ave			46	6907.78	3049.50	46793	56750.26	1609.04
88	0	0	12	994203.48	10.73	716600	1710814.25	1657.4
	3	0.05	12	993615.75	3.62	716600	1710219.37	1637.3
		0.1	12	**993588.82**	1.53	716600	**1710190.37**	1640.8
		0.15	12	993652.6	**0**	716600	1710252.62	1349.4
	5	0.05	12	993615.75	3.62	716600	1710219.37	1639.1
		0.1	12	**993588.82**	1.53	716600	**1710190.37**	1654.5
		0.15	12	993625..18	0.42	716600	1710225.62	1349.3
	7	0.05	12	1014708.7	31.34	**705600**	1720339.87	1638.7
		0.1	12	993635.28	**0**	716600	1710235.25	1638.6
		0.15	12	993657.85	3.35	716600	1710261.25	1353.3
	10	0.05	12	993615.75	3.62	716600	1710219.37	1640.5
		0.1	12	993638.91	1.71	716600	1710240.62	1643.5
Ave			16.86	843481.817	439.78	620127.6	1474773.69	1575.80
150	0	0	12	1106560.81	21.56	**1200000**	2306582.37	1642.2
	3	0.05	13	1038497.03	2.43	1300000	2338499.46	1648.3
		0.1	12	1096512.68	0.31	**1200000**	2296512.99	1647.9
		0.15	12	1104793.7	**0**	**1200000**	2304793.7	1642.8
	5	0.05	12	1100643.5	**0**	**1200000**	2300643.5	1639.5
		0.1	12	1108980.18	5.01	**1200000**	2308985.19	1635.2
		0.15	12	1095116.32	**0**	**1200000**	2295116.32	1573.8
	7	0.05	12	1094878.28	**0**	**1200000**	2294878.28	1649.9
		0.1	12	1091417.19	**0**	**1200000**	2291417.19	1640.1
		0.15	12	1108959.33	**0**	**1200000**	2308959.33	1639.4
	10	0.05	12	**1086525.15**	**0**	**1200000**	**2286525.15**	1631.6
		0.1	12	1108295.16	**0**	**1200000**	2308295.16	1638.5
		0.15	12	1108295.16	1.82	**1200000**	2308296.98	1642.8
Ave			12.07692	1096113.42	2.40	1207692	2303808.12	1636.31

By comparing the costs of the entire supply chain across different scenarios, it becomes evident that the design of the supply chain is sensitive to both the increase in the number of facilities whose capacity can be augmented in the event of a disruption (b) and the magnitude of the capacity increase (a). Across all experimental instances with the same number of nodes, an increase in these parameters leads to a decrease in supply chain costs when there is no possibility of capacity increase (a=0, b=0). Particularly in scenarios with 49 and 50 nodes, the experimental instances with the greatest increase in these parameters exhibit the lowest total cost. Furthermore, in scenarios with 49 nodes, an increase in capacity leads to a reduction in the number of opened facilities, resulting in increased transportation costs. However, in scenarios with 88 and 150 nodes, the lowest total cost did not occur with the highest values for the *b* and *a* parameters. Specifically, in the problem instances with 88 nodes, the minimum cost was observed in two instances: when *b* equals 3 and 5 with a value of 0.1. Similarly, for the scenario with 150 nodes, the minimum cost was observed when b equals 10 and *a* equals 0.05. These results highlight that the highest values for both parameters do not always lead to the lowest supply chain cost. Therefore, sensitivity analysis is crucial for determining the optimal parameter combination.

**Table 8:** Computational results of the proposed RFH algorithm for the test instances with different failure probabilities levels

Node	b	*v*	Lowest						Highest						Variable	
			#	$C_{T}$	$C_{L}$	$C_{F}$	$T_{c}$	Cpu(s)	#	$C_{T}$	$C_{L}$	$C_{F}$	$T_{c}$	Cpu(s)	$Imp_{3}$	$Imp_{4}$
49	0	0	10	546810.9	2.55	702600	1249413.45	21.8	10	507060.74	1102262.85	674800	2284123.59	14.49	0.2	45.41
	3	0.05	8	607998.15	0	566600	1174598.15	8.8	7	605658.74	836955.19	506300	1948913.92	7.4	0.08	39.78
		0.1	8	607998.15	0	566600	1174598.15	8.7	7	605658.74	836955.19	506300	1948913.92	7.1	0.08	39.78
		0.15	8	607998.15	0	566600	1174598.15	8.4	7	605658.74	836955.19	506300	1948913.92	7.1	0.08	39.78
	5	0.05	8	607998.81	0.18	566600	1174598.99	8.3	7	605658.74	836955.19	506300	1948913.92	7.1	-0.01	39.72
		0.1	8	607998.81	0.18	566600	1174598.99	8.7	7	605658.74	836955.19	506300	1948913.92	7.3	0.08	39.78
		0.15	8	607998.15	0	566600	1174598.15	8.9	7	605658.74	836955.19	506300	1948913.92	7.2	0.08	39.78
	7	0.05	8	607998.15	0	566600	1174598.15	8.4	7	605658.74	836955.19	506300	1948913.92	7.1	-0.01	39.72
		0.1	8	607998.81	0.18	566600	1174598.99	8.5	7	605658.74	836955.19	506300	1948913.92	7.2	-0.01	39.73
		0.15	8	607998.15	0	566600	1174598.15	8.2	7	605658.74	836955.19	506300	1948913.92	7.1	0.08	39.78
Ave			8.2	601879.62	0.31	580200	1182079.93	9.87	7.3	595798.94	863485.96	523150	1982434.89	7.91	0.07	40.41
50	0	0	46	6739.83	3095.16	46793	56627.99	20.6	46	972.71	12264.94	46793	60030.65	18.7	-0.31	5.377
	3	0.05	46	6739.85	3095.15	46793	56628	19.7	46	972.71	12264.94	46793	60030.65	17.4	-0.16	5.522
		0.1	46	6739.85	3095.15	46793	56628	19.4	46	972.71	12264.94	46793	60030.65	17.5	-0.3	5.39
		0.15	46	6739.85	3095.15	46793	56628	20.5	46	972.71	12264.94	46793	60030.65	17.4	-0.44	5.252
	5	0.05	46	6739.85	3095.15	46793	56628	20.4	46	972.71	12264.94	46793	60030.65	17.5	-0.16	5.516
		0.1	46	6739.85	3095.15	46793	56628	19.3	46	972.71	12264.94	46793	60030.65	17.1	-0.17	5.506
		0.15	46	6739.85	3095.15	46793	56628	18.7	46	972.71	12264.94	46793	60030.65	16.7	-0.16	5.519
	10	0.5	46	6739.85	3095.15	46793	56628	19.4	46	972.71	12264.94	46793	60030.65	17.3	-0.11	5.563
		0.1	46	6739.85	3095.15	46793	56628	19.5	46	972.71	12264.94	46793	60030.65	17.3	-0.28	5.408
		0.15	46	6739.85	3095.15	46793	56628	19.4	46	972.71	12264.94	46793	60030.65	17.4	-0.08	5.593
Ave			46	6739.85	3095.15	46793	56627.99	19.69	46	972.71	12264.94	46793	60030.65	17.43	-0.22	5.46
88	0	0	11	1073556.88	29.27	656700	1730286.15	141.6	11	943425.67	1932426.43	656700	3532552.1	66.2	1.13	51.57
	3	0.05	11	1068327.55	31.07	656700	1725058.62	109.4	11	943398.25	1932426.43	656700	3532524.68	74.6	0.86	51.59
		0.1	11	1068327.55	31.07	656700	1725058.62	96.1	11	943398.25	1932426.43	656700	3532524.68	63.5	0.86	51.59
		0.15	11	1068327.55	31.07	656700	1725058.62	91.8	11	943398.25	1932426.43	656700	3532524.68	62.2	0.86	51.59
	5	0.05	11	1068327.55	31.07	656700	1725058.62	109.3	11	943398.25	1932426.43	656700	3532524.68	64.9	0.86	51.59
		0.1	11	1068327.55	31.07	656700	1725058.62	127.5	11	943398.25	1932426.43	656700	3532524.68	64.4	0.86	51.59
		0.15	11	1068327.55	31.07	656700	1725058.62	129.8	11	943398.25	1932426.43	656700	3532524.68	64.7	0.86	51.59
	7	0.05	11	1068327.55	31.07	656700	1725058.62	130.2	11	943398.25	1932426.43	656700	3532524.68	60.4	0.27	51.3
		0.1	11	1068327.55	31.07	656700	1725058.62	119.3	11	943398.25	1932426.43	656700	3532524.68	61.6	0.86	51.59
		0.15	11	1068327.55	31.07	656700	1725058.62	122.9	11	943398.25	1932426.43	656700	3532524.68	62.1	0.86	51.59
	10	0.05	11	1068327.55	31.07	656700	1725058.62	112.9	11	943398.25	1932426.43	656700	3532524.68	61.4	0.86	51.59
		0.1	11	1068327.55	31.07	656700	1725058.62	111.2	11	943398.25	1932426.43	656700	3532524.68	68.4	0.86	51.59
		0.15	11	1068327.55	31.07	656700	1725058.62	114.6	11	943398.25	1932426.43	656700	3532524.68	61.2	0.86	51.59
Ave			11	1068729.81	30.93	656700	1725460.74	116.66	11	943400.36	1932426.43	656700	3532526.79	64.28	0.84	51.56
150	0	0	11	1252485.89	206.69	1100000	2352692.58	133.9	11	1045425.2	3028122.9	1100000	5173548.2	186.1	1.96	55.42
	3	0.05	12	1150687.09	215.26	1200000	2350902.35	128.8	11	1045425.2	3028122.9	1100000	5173548.2	206.6	0.53	54.8
		0.1	12	1150687.09	215.26	1200000	2350902.35	124.4	11	1045425.2	3028122.9	1100000	5173548.2	191.1	2.31	55.61
		0.15	12	1150687.09	215.26	1200000	2350902.35	119	11	1045425.2	3028122.9	1100000	5173548.2	197.7	1.96	55.45
	5	0.05	12	1150687.09	215.26	1200000	2350902.35	119.7	11	1045425.2	3028122.9	1100000	5173548.2	191.3	2.14	55.53
		0.1	12	1150687.09	215.26	1200000	2350902.35	121.3	11	1045425.2	3028122.9	1100000	5173548.2	188.8	1.78	55.37
		0.15	12	1150687.09	215.26	1200000	2350902.35	126.4	11	1045425.2	3028122.9	1100000	5173548.2	186.6	2.37	55.64
	7	0.05	12	1150687.09	215.26	1200000	2350902.35	117.4	11	1045425.2	3028122.9	1100000	5173548.2	180.1	2.38	55.64
		0.1	12	1150687.09	215.26	1200000	2350902.35	120.8	11	1045425.2	3028122.9	1100000	5173548.2	196.6	2.53	55.71
		0.15	12	1150687.09	215.26	1200000	2350902.35	125.5	11	1045425.2	3028122.9	1100000	5173548.2	200.2	1.78	55.37
	10	0.05	12	1150687.09	215.26	1200000	2350902.35	116.3	11	1045425.2	3028122.9	1100000	5173548.2	187.6	2.74	55.8
		0.1	12	1150687.09	215.26	1200000	2350902.35	118.6	11	1045425.2	3028122.9	1100000	5173548.2	181.8	1.81	55.38
		0.15	12	1150687.09	215.26	1200000	2350902.35	120.5	11	1045425.2	3028122.9	1100000	5173548.2	186.7	1.81	55.38
Ave			11.92	1158517.77	214.60	1192307.69	2351040.06	122.51	11	1045425.2	3028122.9	1100000	5173548.2	190.86	2.01	55.47

### The analysis of failure probabilities of facilities

In this section, to investigate the effect of considering the same probability of failure on the solutions of the problem, the proposed RFH algorithm is used to solve the experimental instances with two identical failure probability levels: low, and high and the results will be compared with those of found by RFH with variable failure probabilities. For the low, and high levels, the failure probabilities are considered equal to the lowest, and largest values of the failure probability in the main problem, respectively. It is worth noting that the reason for selecting these two levels is to consider the estimation of scenarios in which an optimistic or pessimistic predictor of the failure probabilities is employed. The results are presented in Table 8. In this table, the specifications of instances are again in the first four columns. The next six columns in Table 8 report the results found by the RFH for the problem instances when considering the same low failure probabilities for all facilities, showing the number of opened facilities ($\#$), the total transportation costs ($C_{T}$), the total penalty costs ($C_{L}$), the total fixed facility location cost ($C_{F}$), the total cost of supply chain ($T_{c}$) as well as the Cpu time (in seconds). The next six columns in these tables include the results found by the proposed RFH when considering the same high failure probabilities for all facilities. To evaluate the quality of the solutions found by RFH, table 8 reports in the last two columns two improvement indices, namely $Imp_{3}$, and $Imp_{4}$. $Imp_{3}$ is the percentage improvement of the best cost produced by RFH when the failure probabilities are site-dependent and variables (the results are reported in table 7) from the solution found by the RFH when the failure probabilities are set to the same low failure probabilities for all facilities; it is computed as $100\cdot \frac{T^{Low}_{c} - T_{c}^{site-dependent}}{T_{c}^{Low}}$. $Imp_{4}$ is the percentage improvement of the best cost produced by RFH when the failure probabilities are site-dependent and variables from the solution found by the RFH when the failure probabilities are set to the same high failure probabilities for all facilities; it is computed as $100\cdot \frac{T^{high}_{c} - T_{c}^{site-dependent}}{T_{c}^{high}}$. Note that values of $Imp_{3}$, and $Imp_{4}$ less than zero denote that RFH produces worse cost, and a worse gap than the MIP solver, respectively. In addition, for each set of experimental instances with the same number of nodes, the average value obtained for each column is presented based on the algorithm’s execution time limit in *Ave* rows. It should be noted that in attempting to solve experimental problems with equal failure probabilities, the objective function used in the model is minimized with the same probabilities. However, to ensure a fair comparison with the results obtained by RFH reported in subsection 5.3, the actual value of the objective function for the obtained answer has been calculated by taking into account the facility-dependent failure probabilities.

Reviewing the average values of two improvement indices, namely $Imp_{3}$ and $Imp_{4}$, for objective functions in Table 8, reveals that when our predictions of failure probabilities are pessimistic, and we use the model with the highest failure probabilities to solve the problem instances, the estimated total cost of the supply chain will be much higher than the total supply chain costs found by RFH for the model with site-dependent failure probabilities (reported in table 7 ). In this case, the average improvement rates for sets of problem instances with 49, 50, 88, and 150 nodes are 40.41%, 5.46%, 51.56%, and 55.47%, respectively. Notably, considering the highest values for failure probabilities increases the expected failure costs of facilities. In instances with 49 nodes, this results in a decrease in the average number of opened facilities from 8.2 (see table 7) to 7.3. Additionally, in this set of instances, average penalty costs have also experienced a substantial increase, while average transportation costs have decreased.

On the other hand, if our estimate of the probability of facility failure is optimistic and we use the model with the same failure probabilities set equal to the lowest value, the estimated total cost of the supply chain will again be higher than the actual costs, particularly in problem instances with 49, 88, and 150 nodes, while slightly better solutions were found for the problem instances with 50 nodes. The average improvement rate, $Imp_{3}$, will grow from 0.07 for the set of experimental instances with 49 nodes to 0.84 and 2.01 in the instances with 88 and 150 nodes respectively, which proves the efficiency of the site-dependent model as the size of the problem grows.

From Table 8, it is evident that the computational times required by RFH to solve the models with the same facility failure probabilities are lower than those for the model with site-dependent probabilities. Therefore, where the variance of facility failure probabilities is negligible, adopting the classical model with the same failure probability set to the lowest value is preferable, as it is significantly faster than the site-dependent model introduced in this work.

### Using Heuristic Bounds to Accelerate Exact Solvers

In this subsection, we investigate the possibility of leveraging the upper bounds obtained from RFH as initial solutions for CPLEX, to assess whether CPLEX can derive tighter bounds using these preliminary results. To do this, we first run RFH for the initial level of time limit of 800 seconds, then use its results as a warm start for CPLEX, which is also run for an additional 800 seconds. For a fair comparison, the results of CPLEX are compared with those of RFH when it is run for the second level of the time limit (1600s). Table 9 summarises the results.

In tables 9, the specifications of instances are in the first three columns: the number of nodes, *b*, and *a*. The next five columns in this table report the results found by the CPLEX when used the results of the RFH as the warm start (RFH-MIP), showing the number of opened facilities ($\#$), the lower bound (LB), the upper bound (UB) of the objective function, the optimality gap (Gap) between such two bounds, as well as the Cpu time (in seconds). The next four columns in these tables include the results found by the proposed RFH, in particular, the number of opened facilities ($\#$), the upper bound (UB) of the objective function, the optimality gap (Gap) between such two bounds, as well as the Cpu time (in seconds). In this table, an improvement index, namely *Imp* also calculates the percentage improvement of the best cost produced by RFH from the solution found by the RFH-MIP method (i.e., UB); it is computed as $100\cdot \frac{UB_{(RFH-MIP)}-UB_{RFH}}{UB_{RFH}}$. Note that values of *Imp* less than zero again denote that RFH produces a worse cost, and a worse gap than the MIP solver, respectively. In addition, for each set of experimental instances with same number of nodes, the average value obtained for each column is presented based on the algorithm’s execution time limit in *Ave* rows.

**Table 9:** Computational results of MIP solver, and the RFH algorithm for the test instances when using heuristic bounds

Node		b	*a*	RFH-MIP					RFH				
				#	*LB*	*UB*	Gap	Cpu(s)	#	*UB*	Gap	Cpu(s)	*Imp*
49		0	0	10	1198020.68	1246759.37	3.91	1616.2	10	1246794.75	3.91	1607.8	-0.00
		3	0.05	8	1147710.11	1174598.19	2.29	1438.9	8	1173573.50	2.20	1328.6	0.09
			0.1	8	1146958.63	1174598.17	2.35	1427.1	8	1173573.50	2.27	1327	0.09
			0.15	8	1143679.86	1173573.56	2.55	1428.7	8	1173573.62	2.55	1323.5	-0.00
		5	0.05	8	1136068.82	1173573.57	3.20	1439.3	8	1173573.50	3.20	1329.3	-0.00
			0.1	8	1144214.77	1173573.56	2.50	1428.5	8	1173573.50	2.50	1326	-0.00
			0.15	8	1149416.40	1173573.56	2.06	1429.2	8	1173573.62	2.06	1324.5	-0.00
		7	0.05	8	1136068.82	1173573.57	3.20	1432.3	8	1174598.00	3.28	1605.4	-0.09
			0.1	8	1143286.56	1173573.56	2.58	1429.5	8	1174598.00	2.67	1326.8	-0.09
			0.15	8	1148358.45	1174598.20	2.23	1425.4	8	1173573.50	2.15	1323.8	0.09
	Ave			8.2	1149378.31	1181199.53	2.69	1449.51	8.2	1181100.55	2.68	1382.3	0.01
50		0	0	46	56037.32	56641.53	1.06	1612.2	46	56643.83	1.07	1610.9	-0.00
		3	0.05	46	55844.13	56739.72	1.58	1610.9	46	56700.05	1.51	1609.9	0.07
			0.1	46	56101.61	56671.35	1.00	1611.6	46	56709.72	1.07	1607.6	-0.07
			0.15	46	55980.15	56637.03	1.16	1616.3	46	56725.24	1.31	1608.2	-0.16
		5	0.05	46	56095.51	56647.42	0.97	1610.8	46	56648.86	0.98	1609.5	-0.00
			0.1	46	55990.76	56634.58	1.14	1614.6	46	56647.87	1.16	1609.8	-0.02
			0.15	46	55926.00	56641.37	1.26	1619.1	46	56638.89	1.26	1607.6	-0.00
		10	0.05	46	55914.11	56678.45	1.35	1611.4	46	56660.40	1.32	1608.5	0.03
			0.1	46	55960.07	56637.91	1.20	1616.5	46	56639.64	1.20	1608.7	-0.00
			0.15	46	55981.50	56643.84	1.17	1612.6	46	56797.66	1.44	1609.7	-0.27
	Ave			46	55983.12	56657.32	1.19	1613.6	46	56681.22	1.23	1609.0	-0.04
88		0	0	12.00	1600356.88	1710915.62	6.46	1663.30	12	1710686.87	6.45	1657.40	0.01
		3	0.05	12.00	1599764.78	1797347.88	10.99	1652.60	12	1710162.25	6.46	1637.30	5.10
			0.1	12.00	1599764.78	1797347.88	10.99	1663.90	12	1710162.25	6.46	1640.80	5.10
			0.15	12.00	1599559.99	1797347.88	11.00	1582.50	12	1710163.12	6.47	1349.40	5.10
		5	0.05	14.00	1598962.05	2045671.50	21.83	1659.90	12	1710162.50	6.50	1639.10	19.62
			0.1	12.00	1599559.99	1797347.88	11.00	1665.40	12	1710162.25	6.47	1654.50	5.10
			0.15	12.00	1599676.17	1797347.88	11.00	1614.50	12	1710163.12	6.46	1349.30	5.10
		7	0.05	12.00	1599560.00	1797347.88	11.00	1662.10	12	1710162.50	6.47	1638.70	5.10
			0.1	12.00	1599676.17	1797347.88	11.00	1656.10	12	1710162.12	6.46	1638.60	5.10
			0.15	12.00	1599560.00	1797347.88	11.00	1591.20	12	1710163.12	6.47	1353.30	5.10
		10	0.05	12.00	1599560.00	1797347.88	11.00	1660.60	12	1710162.37	6.47	1640.50	5.10
			0.1	12.00	1599560.00	1797347.88	11.00	1655.00	12	1710162.12	6.47	1643.50	5.10
			0.15	12.00	1599560.00	1797347.88	11.00	1608.90	12	1710163.12	6.47	1351.60	5.10
	Ave			12.15	1599624.68	1809801.06	11.48	1641.23	12.00	1710202.90	6.47	1553.4	5.83
150		0	0	-	-	-	-	-	12	2414217.75	NA	1702.5	NA
		3	0.05	-	-	-	-	-	11	2362812.5	NA	1703.6	NA
			0.1	-	-	-	-	-	14	2466310.5	NA	1724.5	NA
			0.15	-	-	-	-	-	12	2362431	NA	1707.3	NA
		5	0.05	-	-	-	-	-	12	2355155.75	NA	1701.6	NA
			0.1	-	-	-	-	-	14	2466378.5	NA	1704.8	NA
			0.15	-	-	-	-	-	12	2362429	NA	1706.7	NA
		7	0.05	-	-	-	-	-	11	2363040.5	NA	1699.8	NA
			0.1	-	-	-	-	-	14	2466344.75	NA	1704.3	NA
			0.15	-	-	-	-	-	12	2362433.75	NA	1699.2	NA
		10	0.05	-	-	-	-	-	12	2355373.5	NA	1706.4	NA
			0.1	-	-	-	-	-	14	2466311.25	NA	1711.3	NA
			0.15	-	-	-	-	-	12	2362432.5	NA	1700.3	NA
	Ave			-	-	-	-	-	12.46154	2397359.33	NA	1705.6	NA

A comparative evaluation of the RFH and RFH-MIP methods, as presented in Table 9, for problem instances involving 49 nodes indicates that RFH yields better results than RFH-MIP in three out of ten cases. However, the corresponding average improvement (Imp) is only 0.01, suggesting a negligible overall advantage. For instances with 50 nodes, RFH-MIP outperforms RFH in eight out of ten cases, with the average Imp values further substantiating its superior performance in this context. In contrast, for problem instances with 88 nodes, RFH consistently surpasses RFH-MIP across all instances, achieving a notable average Imp value of 5.83, which reflects a significant performance gain. For larger instances involving 150 nodes, while RFH is able to generate a feasible initial solution that can be utilized by CPLEX as a warm start, the RFH-MIP approach fails to produce any results due to CPLEX encountering memory limitations during the solution process.

The results indicate that RFH-MIP tends to produce more competitive solutions for smaller problem instances, as evidenced by its superior performance in the 50-node cases. However, as the problem size increases, RFH demonstrates greater efficiency and robustness, particularly in terms of average processing time and solution feasibility. This suggests that while RFH-MIP may be preferable for small-scale problems, RFH is more suitable for solving larger instances due to its scalability and lower computational demands.

### Computational Impact of Variable Failure Probabilities

In this section, we analyse the complexity of the proposed model with variable failure probabilities for all facilities, as presented in Section 3. To evaluate the impact of this generalisation on computational complexity, we compare the computation times of our model with those of the model proposed in Albareda-Sambola et al. (2017), which assumes constant failure probabilities. For a fair comparison, we apply a uniform failure probability across all facilities, set equal to the average of the variable probabilities used in our model. The MILP formulation was implemented in IBM ILOG CPLEX 22.1 and solved on the same computer, with specifications reported in Subsection 5.2, to solve experimental instances with 49, 50, and 88 nodes. The experimental instances with 150 nodes were excluded from this analysis, as CPLEX was unable to solve either model (with variable or identical failure probabilities) due to memory limitations. The results are presented in Table 10.

As in Table 6, the specifications of the instances are provided in the first four columns of Table 10: the number of nodes, time limits, the limit on the number of facilities whose expected demands exceed their capacities (*b*), and the limit on the expected demand exceeding each facility’s capacity multiplier (*a*). The next five columns show the results obtained by CPLEX for the model with variable failure probabilities, including the number of opened facilities (#), the lower bound (LB), the upper bound (UB) of the objective function, the optimality gap (Gap) between these two bounds (computed as $\frac{UB - LB}{UB}$), and the CPU time (in seconds). The final five columns report the results of the model with identical facility failure probabilities.

**Table 10:** Computational results of the MIP solver for the test instances considering both variable and identical facility failure probabilities

Node	Timelimit	b	*a*	Variable					Identical				
				#	*LB*	*UB*	*Gap*	Cpu(s)	#	*LB*	*UB*	*Gap*	Cpu(s)
49	800	0	0	11	1200046.2	1258960.31	4.68	812.1	10	1259118.07	1259378.14	0.02	802.7
		3	0.05	8	1141179.64	1175356.01	2.91	812.2	8	1191035.00	1191035.00	0	368.4
			0.1	8	1149160.92	1174599.36	2.16	808.5	8	1191035.00	1191035.00	0	395.3
			0.15	8	1143187.49	1173706.81	2.6	807.8	8	1191035.00	1191035.00	0	397.1
		5	0.05	8	1141537.37	1174675.86	2.82	808.1	8	1191035.00	1191035.00	0	349.3
			0.1	8	1141928.82	1174818.75	2.8	807.6	8	1191035.00	1191035.00	0	366.2
			0.15	8	1142511.75	1174991.78	2.76	808.1	8	1191035.00	1191035.00	0	369.9
		7	0.05	8	1140394.6	1175161.04	2.96	818.3	8	1191035.00	1191035.00	0	372.3
			0.1	7	1122268.28	1212795.1	7.46	1163.7	8	1191035.00	1191035.00	0	354.3
			0.15	7	1122268.28	1212795.1	7.46	2008.5	8	1191035.00	1191035.00	0	385.1
	Ave			8.1	1144448.33	1190786.01	3.86	965.5	8.2	1197843.31	1197869.31	0.00	416.1
	1600	0	0	11	1205703.97	1258843.54	4.22	1609.7	10	1259333.12	1259333.12	0	849.6
		3	0.05	8	1144520.54	1174616.95	2.56	1600	8	1191035.00	1191035.00	0	368.4
			0.1	8	1151211.66	1174604.78	1.99	1600	8	1191035.00	1191035.00	0	395.3
			0.15	8	1145806.81	1174599	2.45	1600	8	1191035.00	1191035.00	0	397.1
		5	0.05	8	1140661.38	1175422.73	2.95	1600	8	1191035.00	1191035.00	0	349.3
			0.1	8	1144647.87	1174626.38	2.55	1600	8	1191035.00	1191035.00	0	366.2
			0.15	8	1145436.72	1174608.26	2.48	1600	8	1191035.00	1191035.00	0	369.9
		7	0.05	8	1145018.15	1174657.49	2.523	1604.4	8	1191035.00	1191035.00	0	372.3
			0.1	8	1144531.28	1174652.5	2.56	1609.1	8	1191035.00	1191035.00	0	354.3
			0.15	8	1145705.07	1174603.02	2.46	1608.4	8	1191035.00	1191035.00	0	385.1
	Ave			8.3	1151324.35	1183123.47	2.67	1603.2	8.2	1197864.81	1197864.81	0	420.8
50	800	0	0	46	56003.58	56871.67	1.53	809.6	46	56661.32	56807.03	0.26	803.9
		3	0.05	46	55403.17	56721.39	2.32	809.5	46	56661.32	56803.47	0.25	803.6
			0.1	46	55229.51	56756.71	2.69	809.1	46	56661.32	56814.81	0.27	803.5
			0.15	46	55320.87	56743.64	2.51	810.3	46	56661.32	56817.78	0.27	803.6
		5	0.05	46	55311.72	56738.91	2.51	809.7	46	56661.32	56807.03	0.26	804.2
			0.1	46	54160.57	56787.98	4.63	810.5	46	56661.32	56813.96	0.27	803.2
			0.15	46	55443.38	56714.66	2.24	809.7	46	56661.32	56814.81	0.27	803.5
		10	0.05	46	55606.08	56676.75	1.89	809.4	46	56661.32	56807.03	0.26	803.5
			0.1	46	54160.6	56820.92	4.68	810.1	46	56661.32	56807.03	0.26	803.7
			0.15	46	55421.02	56712.34	2.27	809.6	46	56661.32	56808.89	0.26	803.7
	Ave			46	55206.05	56754.50	2.73	809.8	46	56661.32	56810.18	0.26	803.6
	1600	0	0	46	56031.87	56659.07	1.11	1600	46	56802.64	56802.64	0.0	1448.8
		3	0.05	46	55454.68	56651.93	2.11	1600	46	56802.64	56802.64	0.0	1465.9
			0.1	46	55386.58	56685.8	2.29	1600	46	56802.64	56802.64	0.0	1448.1
			0.15	46	55411.04	56657.94	2.2	1600	46	56802.64	56802.64	0.0	1416.7
		5	0.05	46	55325.26	56670.09	2.37	1600	46	56802.64	56802.64	0.0	1448.7
			0.1	46	54194.36	56675.3	4.38	1600	46	56802.64	56802.64	0.0	1451.1
			0.15	46	55535.37	56658.54	1.98	1600	46	56802.64	56802.64	0.0	1418.1
		10	0.05	46	56141.02	56665.94	0.9	1600	46	56802.64	56802.64	0.0	1459.1
			0.1	46	54216.51	56713.02	4.4	1600	46	56802.64	56802.64	0.0	1441.6
			0.15	46	55521.29	56658.81	2.01	1600	46	56802.64	56802.64	0.0	1455.3
	Ave			46	55321.80	56669.64	2.38	1600.0	46	56802.64	56802.64	0.00	1446.4
88	800	0	0	12	1599804.08	1802793.05	11.26	868.8	0	0.00	42545585.86	100.00	864.9
		3	0.05	12	1600370.40	1836418.90	12.85	866.1	0	0.00	42545585.86	100.00	892.6
			0.1	12	1600441.40	1836170.21	12.83	872.6	0	0.00	42545585.86	100.00	831.5
			0.15	12	1600108.70	1807689.91	11.48	864.4	0	0.00	42545585.86	100.00	831.1
		5	0.05	12	1600370.40	1836418.90	12.85	864.6	0	0.00	42545585.86	100.00	834.3
			0.1	12	1600158.84	1835177.55	12.81	863.3	0	0.00	42545585.86	100.00	835.4
			0.15	12	1600108.70	1807689.91	11.48	863.5	0	0.00	42545585.86	100.00	890.6
		7	0.05	12	1600370.40	1836418.90	12.85	864.6	0	0.00	42545585.86	100.00	852.3
			0.1	12	1600570.03	1835177.55	12.78	872.8	0	0.00	42545585.86	100.00	848.6
			0.15	12	1600353.15	1807689.91	11.47	863.2	0	0.00	42545585.86	100.00	833.1
		10	0.05	12	1600475.38	1836418.90	12.85	865.8	0	0.00	42545585.86	100.00	812.2
			0.1	12	1600371.93	1835177.55	12.79	867.9	0	0.00	42545585.86	100.00	819.1
			0.15	12	1600353.15	1807689.91	11.47	866.3	0	0.00	42545585.86	100.00	827.3
	Ave			12	1600296.66	1824687.01	12.29	866.5	0	0.00	42545585.86	100	844.1
	1600	0	0	12	1604989.1	1802793.05	10.97	1700	0	0.0	42545585.86	100	1640.1
		3	0.05	12	1606068.45	1836418.9	12.54	1700	0	0.0	42545585.86	100	1640.3
			0.1	12	1606042.54	1836170.21	12.53	1700	0	0.0	42545585.86	100	1674.8
			0.15	12	1605224.12	1766019.84	9.1	1700	0	0.0	42545585.86	100	1694.1
		5	0.05	12	1606068.45	1836418.9	12.54	1700	0	0.0	42545585.86	100	1691.3
			0.1	12	1605798.94	1835177.55	12.5	1700	0	0.0	42545585.86	100	1655.4
			0.15	12	1605252.9	1766019.84	9.1	1700	0	0.0	42545585.86	100	1640.6
		7	0.05	12	1606068.45	1767449.65	9.13	1700	0	0.0	42545585.86	100	1684.5
			0.1	12	1605798.94	1835177.55	12.49	1700	0	0.0	42545585.86	100	1640.7
			0.15	12	1605252.9	1766019.84	9.1	1700	0	0.0	42545585.86	100	1675.6
		10	0.05	12	1606068.45	1836418.90	12.54	1700.0	0	0.00	42545585.86	100.00	1684.3
			0.1	12	1605586.33	1835177.55	12.51	1700.0	0	0.00	42545585.86	100.00	1632.9
			0.15	12	1605224.12	1807689.91	11.20	1700.0	0	0.00	42545585.86	100.00	1657.6
	Ave			12	1605649.51	1809765.51	11.25	1700.0	0	0.00	42545585.86	100.00	1662.5

An examination of the results presented in Table 10 indicates that, for instances with 49 nodes, the model assuming identical failure probabilities was solved to optimality within 849.6 seconds. In contrast, the corresponding instances incorporating variable failure probabilities could not be solved to optimality within the CPLEX time limit of 1600 seconds. A similar pattern is observed for instances with 50 nodes: CPLEX successfully solved all instances under the identical failure probability assumption in less than 1465.9 seconds, whereas none of the instances with variable failure probabilities reached optimality within the imposed time limit.

Interestingly, for larger instances involving 88 facilities, the model with variable failure probabilities outperformed the identical-probability model both in terms of solution quality and computational efficiency. The average optimality gap for the identical probability model was 100%, indicating that no feasible solution was found within the time limit. In contrast, the average gap for the variable failure probability model was 12.29% when the computation time limit was set to 800 seconds, and it further decreased to 11.25% when the time limit was extended to 1600 seconds.

These findings suggest that, while the assumption of identical failure probabilities may simplify the model formulation, it does not necessarily lead to improved computational performance or better-quality solutions, particularly for larger instances.

## Conclusion

The reliable capacitated fixed-charge facility location problem is addressed in this paper. A mixed-integer programming model is proposed for the defined problem which minimises the total cost of locating the facilities and expected disruption costs. Since the proposed model is an extension of the well-known NP-hard fixed-charge facility location problem, finding the optimal solution may lead to excessive computational times; for this reason, a relax-and-fix heuristic is developed. To evaluate the performance of the proposed heuristic, a set of experimental instances was selected from the literature and solved. The comparison between the results of RFH and the ones found by the IBM ILOG Cplex 22.1 MIP solver shows the effectiveness of the proposed algorithm. The performance of the proposed model was compared with classical models on the generated instances and the results confirm the effectiveness of the proposed method. Additionally, sensitivity analysis of expected facility capacity revealed that increasing the number and extent of capacity upgrades generally reduces total supply chain costs, although the optimal parameter combination depends on the scenario, emphasising the need for tailored capacity planning. The failure probability sensitivity analysis showed that models assuming uniform failure probabilities, either high or low, tend to overestimate supply chain costs compared to the site-dependent model. While uniform-probability models solve faster, the site-dependent approach provides more cost-effective and accurate solutions, particularly for larger instances. The heuristic bound analysis further demonstrated that RFH-generated upper bounds can effectively accelerate CPLEX, especially for large-scale problems. Although RFH-MIP performs better for smaller instances, RFH offers superior scalability and efficiency for larger cases. Finally, the computational impact analysis of variable failure probabilities revealed that, despite increased computational effort for smaller problems, this generalization enhances solution quality and feasibility in larger instances, indicating that assuming identical failure probabilities may unnecessarily limit model performance.

Several possible future developments exist for this work. First, generalizing the model from single products to multi-products is a direction for future research. Second, it is possible to overcome the use of MIP solvers in the RFH approach by using heuristic methods to solve models generated in phase 2 and to design more powerful solution approaches. Optima-seeking algorithms such as the column generation or benders’ decomposition approaches may be proposed to solve the problem in a more reasonable amount of time.

## Footnotes

1 The whole data set can be downloaded from the website: http://people.brunel.ac.uk/~mastjjb/jeb/orlib/pmedcapinfo.html.
